# Within-host population structure, migration, and parallel adaptive evolution of *Pseudomonas aeruginosa* in cystic fibrosis lung disease

**DOI:** 10.1128/msystems.00238-26

**Published:** 2026-08-25

**Authors:** David Ritz, Michelle E. Clay, Ted Kim, Rachel D. Van Gelder, Jayadevi H. Chandrashekhar, Alan J. Collins, Alix Ashare, Jay Goddard, Kenneth B. Hoehn, Daniel Schultz, Rachel J. Whitaker, Deborah A. Hogan

**Affiliations:** 1 Department of Microbiology and Immunology, Geisel School of Medicine at Dartmouth, Hanover, New Hampshire, USA; 2 Department of Microbiology, University of Illinois Urbana-Champaign 14589, Urbana, Illinois, USA; 3 Carl R. Woese Institute for Genomic Biology, University of Illinois Urbana-Champaign 14589, Urbana, Illinois, USA; 4 Section of Pulmonary and Critical Care Medicine, Dartmouth-Hitchcock Medical Center 22916, Lebanon, New Hampshire, USA; 5 Department of Molecular and Systems Biology, Geisel School of Medicine at Dartmouth, Hanover, New Hampshire, USA; University of Birmingham, Birmingham, United Kingdom

**Keywords:** *Pseudomonas aeruginosa*, cystic fibrosis, evolution, lung, migration, phylogeny, antibiotic resistance, alginate

## Abstract

*Pseudomonas aeruginosa* infections in adults with cystic fibrosis (CF) are comprised of heterogeneous populations, most often tracing ancestry back to a single recent common ancestor. What is not clear is the physical spatial structure within the lung infection population, its stability over time, and whether this physical structure leads to different evolutionary trajectories in different adaptive environments. To compare the *P. aeruginosa* populations across a single lung, we performed whole genome sequence analyses of 450 isolates recovered from lavage samples of the three different lobes of the right lung from a person with mild-to-moderate CF lung disease at three time points over the course of ~1.5 years. We found that isolates fell into five distinct phylogenetic lineages with evidence for repeated translocation of isolates from different lineages across lobes and loss-of-function mutations in *lasR* and *mucA* were present in all 450 isolates. The well-resolved phylogenetic analyses revealed a structured population in which we find the coexistence of a slowly evolving lineage and more rapidly evolving lineages. There is also support for numerous migration events. Furthermore, strong evidence for parallel adaptive mutations in multiple genes revealed distinct evolutionary paths affecting mucoid phenotypes and genetic variation in antibiotic resistance-associated pathways across coexisting populations within a single individual over time. These results provide an example of within-host evolution leading to microheterogeneity that may be useful to consider in future study of infection metapopulations dynamics over the course of chronic infection.

**IMPORTANCE:**

Individuals with cystic fibrosis (CF) commonly have chronic lung infections that contain clonally derived *Pseudomonas aeruginosa* populations with genotypic and phenotypic diversity. This study describes a substantial data set containing 450 isolates from different lobes of the right lung across three time points from an individual with mild-to-moderate CF lung disease. Some regional enrichment for specific lineages with parallel mutations among individual lobes of the lung was observed, but longitudinal analysis also demonstrated that compartmentalization is not strictly maintained and that isolates migrate between lobes of the lung over time. Perspectives on within lung evolution will be important for understanding the pathogen populations in chronic respiratory infections in CF and other diseases.

## INTRODUCTION

Pulmonary infection by *Pseudomonas aeruginosa* is known to correlate with increased lung disease severity and heightened mortality in individuals with cystic fibrosis (CF) (1–3). Parallel selection for mutations in the same genes within and between CF infections supports the hypothesis that *P. aeruginosa* adapts to the CF lung environment over time. For example, frequently observed loss-of-function mutations in the *lasR* gene, which encodes a quorum sensing regulatory transcription factor (4–7), result in advantageous phenotypes including increased microoxic fitness and biofilm adaptation (8, 9), improved growth (4), and modulation of host responses (10–12). Another set of commonly mutated genes are those involved in the regulation of the production of alginate (e.g., *mucA* and *algU*), an exopolysaccharide that plays a protective role against a variety of environmental stressors (13–17). Other common mutations include loss-of-function mutations in *mexZ* and other regulators involved in drug resistance, presumably because CF patients often receive frequent courses of antibiotics (18).

Population scale studies of *P. aeruginosa* and other microbes associated with chronic infections have clearly established that phenotypically and genotypically heterogeneous populations persist in the CF lung over time (19–22). Sequencing of *P. aeruginosa* isolates reveals that heterogeneity among strains often, but not always, converges to a single common ancestor, indicating emergence from a clonally derived population of *P. aeruginosa* (3, 5, 23–32). Parallel independent evolution events have been observed in different lineages in a single patient (5), suggesting the presence of a complex physically structured environment in the human lung. Lieberman et al. (19) found similar preservation of diversity over time in *Burkholderia dolosa* and suggested that competing adaptive mutations might preserve diversity through clonal competition in addition to spatial structure. An understanding of the spatial and temporal differences within single chronic infections will aid in devising suitable treatment regimes. Toward increasing this understanding, Jorth et al. (23) used lung explants from CF subjects with severe disease to provide phenotypic and genotypic evidence for the physical separation of *P. aeruginosa* populations and their independent evolution. While this study clearly supports the model that physical structure limits migration between lobes, it is not clear if subpopulations are stably associated with specific regions over longer periods of time.

To contribute to the understanding of the evolutionary dynamics of *P. aeruginosa* over space and time, we examined *P. aeruginosa* populations derived from bronchoalveolar lavage (BAL) fluid. Four hundred and fifty isolates from BAL fluid collected from different lobes of the right lung from the same individual with mild CF lung disease at three time points were analyzed by whole-genome sequencing. Visualization of the phylogenetic relationships among these isolates showed the presence of five major clusters. Members of each cluster were enriched in specific lobes at a single time point, but there was evidence for migration between lobes between time points followed by expansion of specific subpopulations. Furthermore, there were differences in mutation rates in different regionally localized subpopulations. While some genetic characteristics associated with CF, such as nonsense mutations in *lasR* and *mucA* genes, were observed in all isolates, there were many instances of parallel evolution events associated with alginate production and drug resistance. Together, these data reveal a dynamic, spatially structured population in which migration and parallel adaptation collectively shape within-host *P. aeruginosa* evolution over time.

## RESULTS

### Characterization of reference genome from a time point 1 isolate

**Fig 1:**
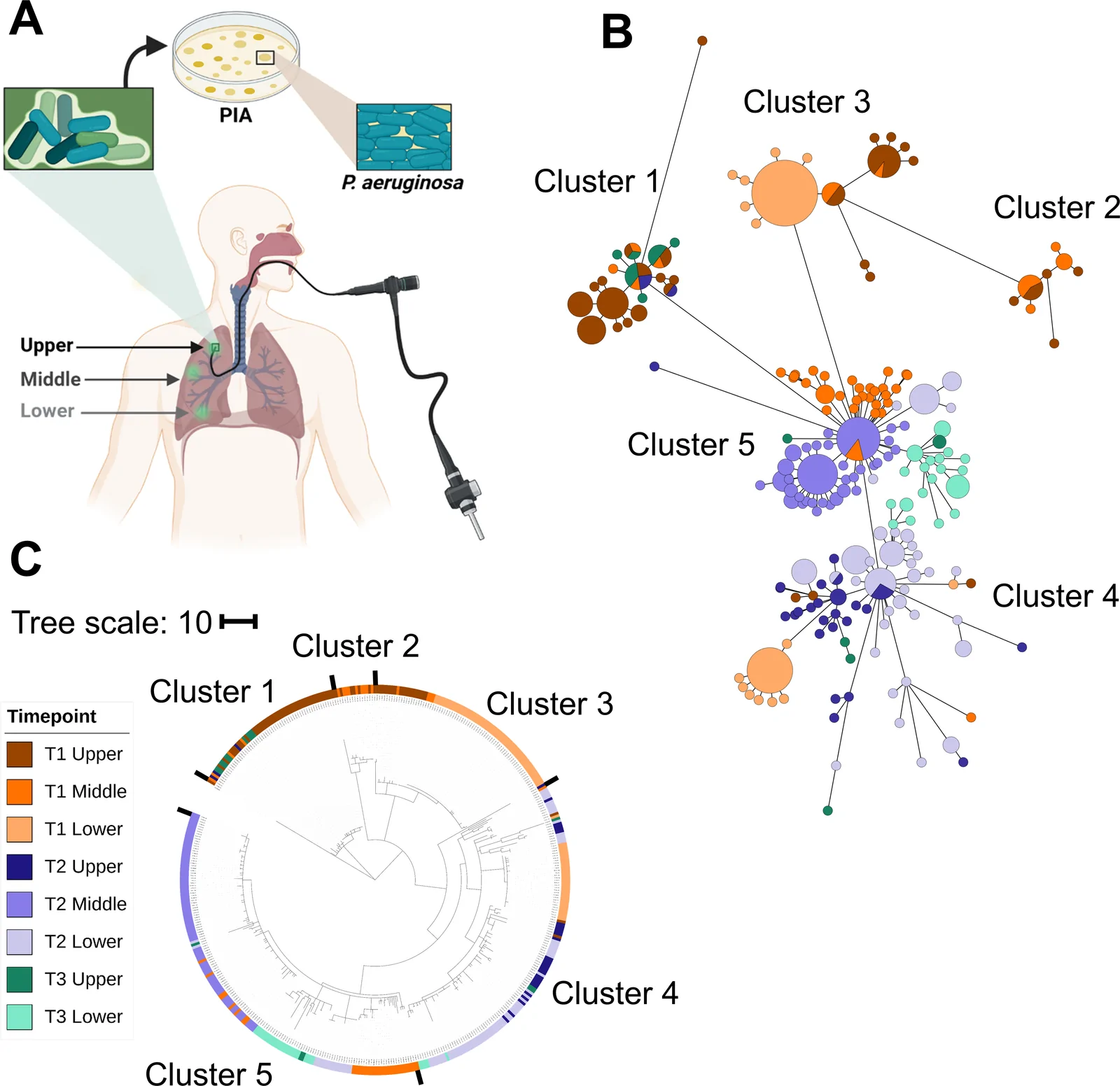
Diverse *P. aeruginosa* strains were isolated from the CF subject’s right lung. (**A**) The methodology for collection of bronchoalveolar lavage (BAL) fluid from the upper, middle, and lower lobes of the subject’s right lung at three time points and subsequent isolation of *P. aeruginosa*. (**B**) GrapeTree represents relationships between sets of identical haplotypes. The size of the grape circle represents the proportion of strains with that haplotype from each sample, and the edges connect haplotypes. The length of the edges represents the number of SNPs between nodes. The three time points are time point 1, time point 2 (151 days later), and time point 3 (353 days after time point 2). (**C**) Rooted circular phylogenetic tree of 450 BAL isolates that includes the ST499 outgroup. The colored bars indicate the isolates’ origin and time points of the BAL. The branch length is equivalent to the number of detected SNPs.

*P. aeruginosa* isolates (*n* = 527) were obtained from BAL samples collected from the right upper, middle, and lower lobes (RUL, RML, and RLL) of an individual with mild-to-moderate CF lung disease at three time points: time point 1, time point 2 (151 days later), and time point 3 (353 days after time point 2) (Fig. 1A). The subject was an adult male with CF, 30–35 years of age at the time of sampling, with an average lung function of 94% predicted forced expiratory volume (FEV1) across the study period. During pulmonary exacerbations, FEV1 declined to approximately 75%. Clinical sputum cultures documented chronic colonization by both mucoid and non-mucoid *P. aeruginosa* for more than 8 years prior to the first BAL collection. Throughout the study period, the subject received multiple antimicrobial treatments, including azithromycin, ceftazidime, tobramycin, ciprofloxacin, vancomycin, piperacillin-tazobactam (Zosyn), doxycycline, and trimethoprim-sulfamethoxazole (Bactrim) as described in detail below.

To study the population structure and distribution of genotypes over time, the 527 strains were sequenced with short-read Illumina technology. To identify single-nucleotide polymorphisms (SNPs) that were unfixed in the population, reads were aligned to an internal reference genome created by sequencing the DNA from isolate T1_LL_B5 using long-read Nanopore technology with polishing using Illumina short read sequences. All isolates belonged to multilocus sequence type (MLST) ST499, consistent with a single common infection origin. Isolate T1_LL_B5 harbored approximately 3,000 SNPs relative to a publicly available genome for another ST499 strain (ERR364087). The ST499 comparator and T1_LL_B5 sequences were more closely related to the epidemic strain LESB58 than to the commonly used laboratory strains PAO1 and PA14 (Fig. S1).

### Detection of variable SNPs in the single-subject isolate collection

After read normalization and alignment of the 527 isolates to the T1_LL_B5 reference genome, variable positions were identified across isolates using breseq (33). Furthermore, both genomes and positions were then filtered to exclude low confidence data using read depth and consensus score metrics as described in detail in Materials and Methods. The 450 isolate genomes that remained after filtering included 45 to 201 isolates per time point and 121 to 196 isolates per lobe (Table 1). Across these strains, there were 663 high confidence variable SNPs (Data set S1) which included 401 non-synonymous SNPs, 198 synonymous SNPs, and 64 intergenic SNPs. In addition, insertions and deletions (indels) were recorded across the collection (Data set S2).

**TABLE 1:** *P. aeruginosa* isolated from the right lung of a person with CF^*a*^

Time point	No. of isolates		
	Upper lobe	Middle lobe	Lower lobe
T1	73	52	85
T2	32	81	82
T3	16	–^*b*^	29

^*a*^ Data stratified by the number of isolates per lung lobe used in the analyses.

^*b*^ “–” indicates that no sample was collected for the given region at the indicated time point.

We first assessed the phylogenetic relatedness of the population using an unrooted grape tree which facilitates the visualization of phylogenetic relationships in the context of metadata such as location and time point (Fig. 1B). We observed the population resolved into five major “clusters,” with isolates from the three time points and the three sampled lung regions within each cluster. The clusters are also indicated on a phylogenetic tree shown in Fig. S2. In a rooted phylogenetic tree (Fig. 1C) that included the ST499 comparator as an outgroup, we observed that the isolates that predominated in the upper lobe within cluster 1 were most closely related to the outgroup. We also observed enrichment of derived lineages within specific regions at each time point as well as strains from different coexisting within regions suggesting migration among lobes.

### Isolates have heterogeneous evolution rates and migration patterns

**Fig 2:**
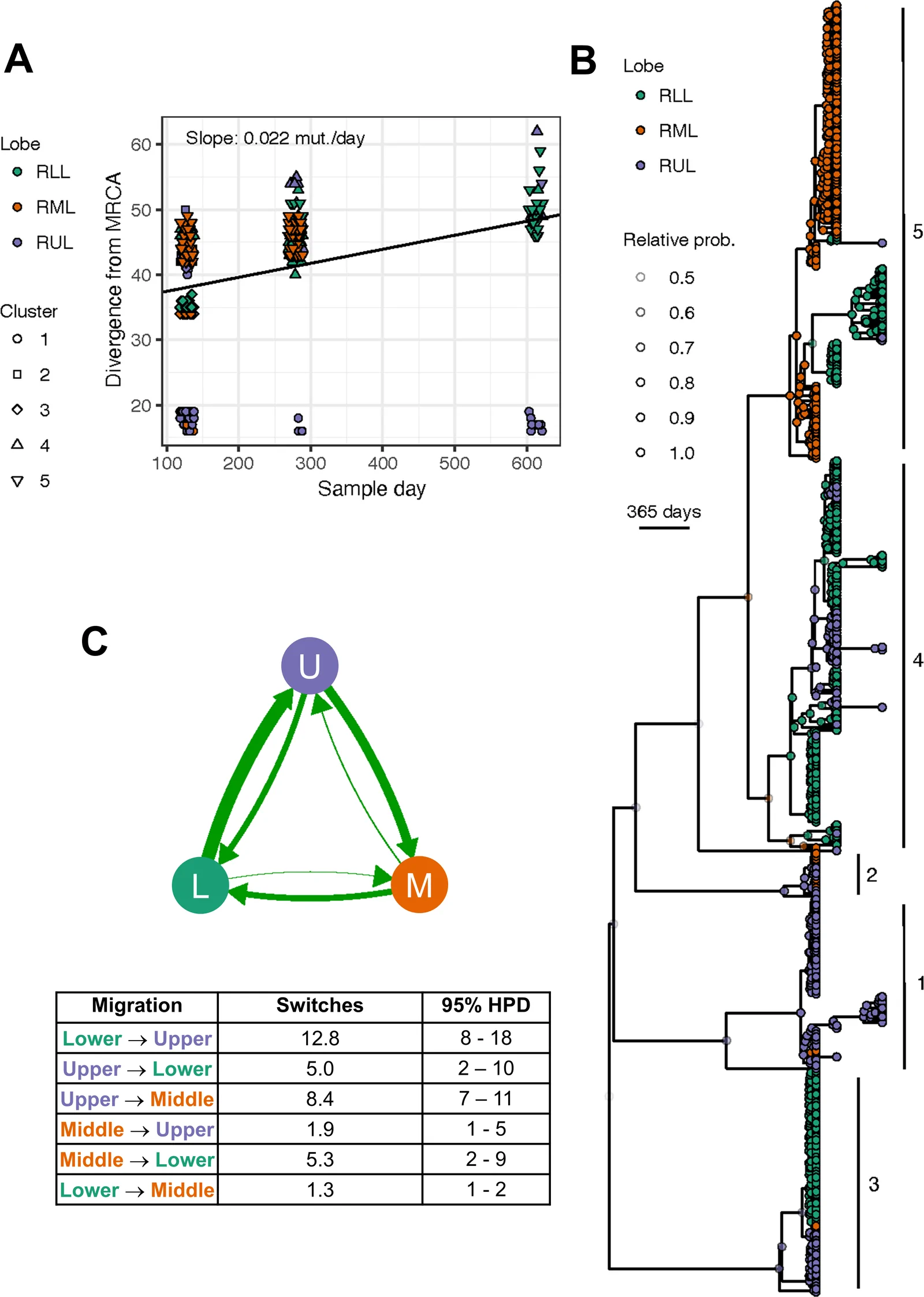
Migration and mutational rates depend on lung lobe. (**A**) Estimated number of migration events between lobes along time trees. Arrow widths are proportional to the mean number of switches (parent nodes with different lobes than child nodes) in each direction along trees sampled from the posterior distribution. The mean number of migrations and their corresponding 95% highest posterior density (HPD) interval are included as a table. (**B**) Time-resolved phylogenetic tree (maximum clade credibility tree) of the CF isolates. Internal nodes are labeled by their estimated location and shaded by its posterior probability. Tips are further labeled by cluster number. (**C**) Root-to-tip regression of genetic divergence of each isolate from the recent common ancestor (MRCA) compared to sample date. Horizontal jitter added to reduce overplotting. The slope of this regression line is the estimated average rate of evolution over the sample interval. The color code is not consistent between this figure and the other figures.

We used the phylogenetic analysis of isolate genomes to reveal dynamics of *P. aeruginosa* evolution and migration within the right lung. If *P. aeruginosa* was evolving over time, isolates at T3 would be more genetically diverged from the most recent common ancestor than isolates at T1 (Fig. 1C). To estimate the average rate of this evolution, we used a root-to-tip regression of genetic divergence against sample date (Fig. 2A) (34). The slope of this regression line indicated an overall rate of ~0.02 mutations per day across the study, significantly greater than zero when assessed using a permutation test (*P* < 1 × 10^−4^). This rate of evolution was not uniform, however. For example, isolates within cluster 1 showed no increase in divergence and, therefore, no evidence of evolution over the 485 days between T1 and T3 (Fig. 2A). These custer 1 strains were the least diverged from the most recent common ancestor and were predominantly localized to the upper lung lobe. These results indicate that *P. aeruginosa* measurably evolved during the study, but that this rate of evolution was heterogeneous among regions and strains within the right lung.

To link the spatial and temporal evolution of *P. aeruginosa* within this subject’s right lung, we inferred time-resolved phylogenies from the CF isolate genomes (Fig. 2B). To do so, we used an uncorrelated lognormal relaxed molecular clock model implemented in BEAST2 (35, 36). We estimated the date of the most recent common ancestor of all sampled isolates as ~4.1 years prior to T1 (95% HPD: 1.8–7.2 years), indicating years of chronic infection compatible with the initial *P. aeruginosa* infection beginning more than 8 years prior to the study. Major genetic clusters 1–5 were identifiable within the maximum clade credibility tree (Fig. 2B). To infer patterns of migration among lobes, we modeled location as a discrete trait using a continuous time Markov model (see Materials and Methods) (35, 37). Across the trees sampled from the posterior, we observed a mean of 32.9 pairs of parent/child nodes where the child node had a different location from the parent, representing a migration event between lobes (Fig. 2C). These switches most frequently occurred from the lower to upper lobe, followed by upper to middle, and middle to lower. Meanwhile, migration events in the opposite directions were much less frequent. These results suggest a cyclical pattern of migration in which *P. aeruginosa* lineages move from the upper lobe through the middle and lower lobes and subsequently return to the upper lobe.

Pairwise fixation index analysis (*F*_ST_), which combines both genetic distance and abundance in all pair wise comparisons between samples, also revealed dynamic spatial and temporal restructuring of the bacterial population across lung lobes (Table S1). For the most part samples are highly differentiated (*F*_ST_ > 0.1) likely showing the clonal nature of local growth captured by BAL sampling. However, the few pairs of samples with very low differentiation (<0.07) supported the evidence for mixing between regions over time. At T1, moderate differentiation among upper, middle, and lower lobes (FST ≈ 0.35–0.37) indicated spatial compartmentalization, consistent with partially isolated intra-lung niches. At T2, this structure was dramatically altered: the upper and lower lobes became highly similar (FST = 0.069), suggesting substantial bacterial mixing or redistribution between these regions. In contrast, the middle lobe at T2 was strongly differentiated from both contemporaneous lobes (FST = 0.60–0.71). By T3, spatial structure re-emerged (T3 upper vs lower FST = 0.477), and notably, the T3 upper lobe population was nearly identical to the T1 upper lobe population (FST = 0.058), suggesting persistence or re-expansion of the slow growing ancestral lineage following the transient T2 restructuring event. It is worth noting that intermediate *F*_ST_ values were observed between the middle lobe at T1 and both the lower and upper lobes at future time points suggesting there was some migration from middle lobe in both directions over time while the middle lobe is dominated by a single type. Without a T3 sample for the middle lobe, the impact of mixing in this region was not assessed. Together, these data support a model in which intra-lung bacterial migration is episodic rather than constant, with temporary breakdown of spatial barriers.

### Predicted adaptive mutations within the population

The evolution of *P. aeruginosa* during the infection resulted in mutations in several mechanisms known to be involved in adaptation to the human host. Missense and nonsense mutations were found in 333 unique PAO1 ORFs, 91% of which were only mutated in single strains. Twenty-two genes had mutations in two or more lineages, indicating parallelism and potentially adaptive evolution (Data set S1). In this section, we focus on specific pathoadaptive loci impacting phenotypic characteristics that have been well characterized and are medically relevant.

#### A *lasR* mutation was fixed in the population

An analysis of the colony morphologies of the isolates found that subsets of the population had a sheen due to the accumulation of 2-heptyl-4-hydroxyquinoline (HHQ), which is very common in strains with loss-of-function mutations in *lasR* (4). While *lasR* was not identified as a gene with unfixed SNPs in the population, comparison of the *lasR* gene sequence in all the isolates to the *lasR* sequence from strain PAO1 found that all 450 isolate genomes contained a nonsense mutation at amino acid Gln45 due to a C135T nucleotide change in the *lasR* gene which led to premature termination. These data suggest that a *lasR* mutation was either present in the original infecting strain or had come to dominate the population in the right lung prior to our studies. As not all isolates had the *lasR* colony phenotype, we searched for mutations in other genes that could impact HHQ production. We found only one isolate with a high confidence missense mutation in *pqsE*, which participates in quorum sensing regulation via effects on RhlR, but no SNPs in other *pqs* genes (Data set S1). Future studies would be required to determine how other loci mutated in this study affect HHQ accumulation and *lasR* phenotypes.

#### Evolution and regional differences of the mucoid phenotype

**Fig 3:**
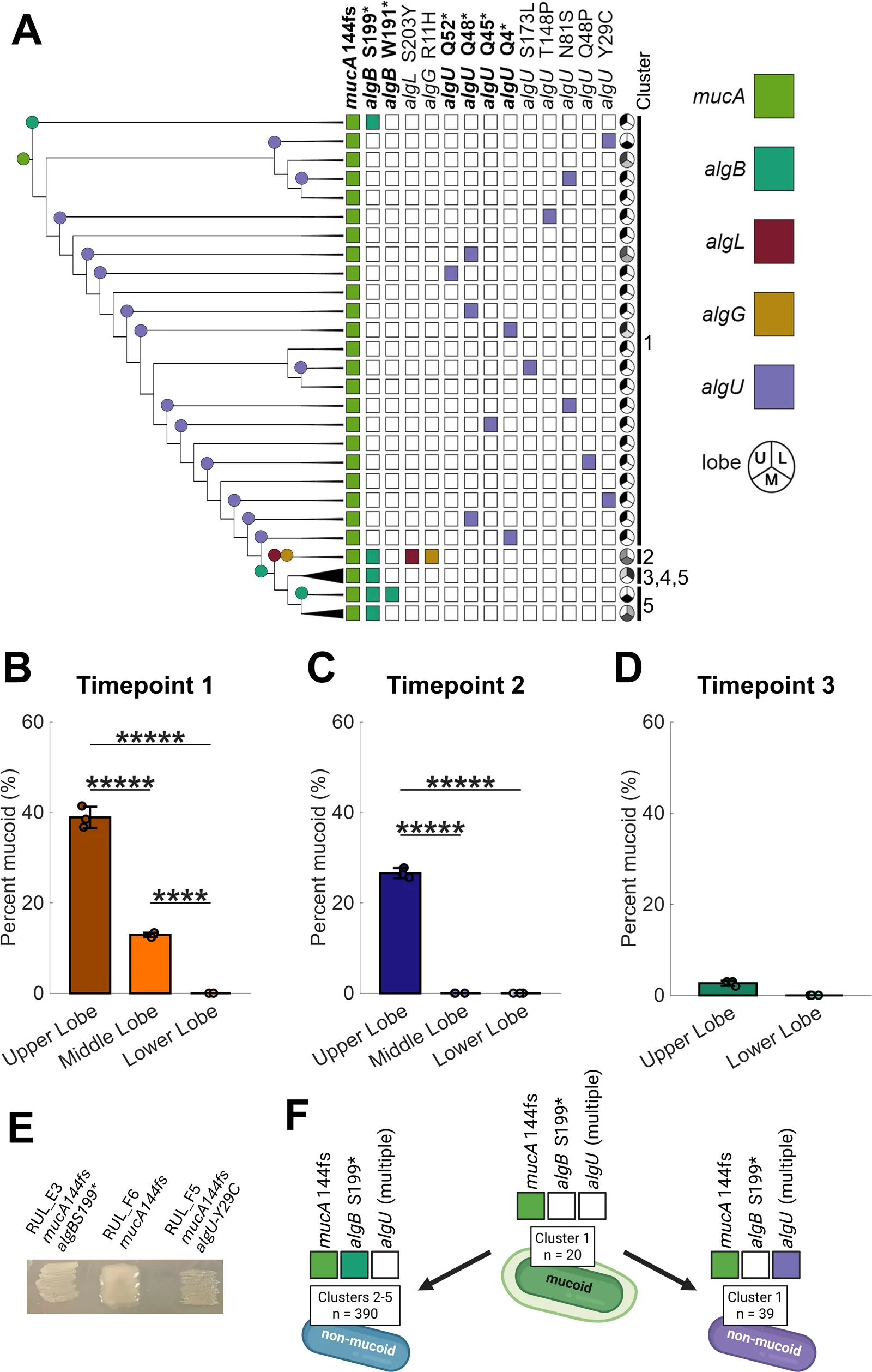
Adaptive mutations cause a variable mucoid phenotype. (**A**) Mutations affecting alginate production are marked in the phylogeny tree of the *P. aeruginosa* isolates. Adjacent strains with identical mutations were collapsed into the same leaf for clarity, with the size of the wedges representing the number of isolates collapsed into each leaf. Colored dots represent where each mutation likely occurred in the phylogeny tree. Cluster numbers are adjacent to the lobe pie graphs. Bold *x*-axis labels represent nonsense or frameshift mutations likely leading to loss of protein function. (**B–D**) The mucoid phenotype was quantified for strains isolated at (**B**) time point 1, (**C**) time point 2, and (**D**) time point 3, with comparisons between the isolate origins. Asterisks denote significance levels for pairwise comparisons between groups: *****P < 10^−5^, ****P < 10^−4^, ***P < 10^−3^, **P < 10^−2^, and *P < 0.05. (**E**) Representative mucoid patches of isolates with either a *mucA* frameshift and *algB* nonsense mutation, only a *mucA* frameshift mutation, or a *mucA* frameshift and *algU* mutation, respectively. (**F**) A frameshift mutation in *mucA* after amino acid 143 is fixed in the population and confers an alginate-producing phenotype in cluster 1 strains. Subsequent mutations in *algU* or *algB* restore the non-alginate phenotype. One outlier strain from clade 1, which carries the *algB* S199* mutation, is not represented in this schematic and corresponds to the first row in Fig. 3A.

We observed that a subset of isolates exhibited a mucoid phenotype associated with increased production of the exopolysaccharide alginate. All 450 isolate genomes carried an identical mutation in *mucA* consisting of a single-nucleotide deletion after amino acid position 143 (Fig. 3A). This frameshift (fs) altered residues 144–146 from Ala-Pro-Gly to Arg-Arg-Arg and introduced a premature stop codon (*mucA*144fs), resulting in truncation of the MucA protein. Loss-of-function mutations in the MucA anti-sigma factor are well known to activate alginate production due to increased AlgU activity (38). Additional mutations were observed in other components of the alginate biosynthetic pathway. Sixteen isolates from the right upper and middle lobes at time point 1 formed a distinct group carrying mutations in both *algG* (R11H) and *algL* (S203Y) (Fig. 3A), suggesting alternative genetic routes to modulate alginate production.

Regional and temporal differences in mucoidy were evident across the isolate collection. Mucoid isolates were most prevalent in samples from the right upper lobe, and the overall frequency of mucoidy declined over the course of the study (Fig. 3B through D). In mucoid strains with low MucA activity, subsequent mutations in *algU* can cause reversion from mucoid to the non-mucoid phenotype (39). Consistent with this, our analysis of unfixed mutations identified nine non-synonymous mutations in *algU*, all confined to cluster 1 isolates, including four nonsense mutations and five missense substitutions (Fig. 3A). In contrast, *algU* remained functional in all other isolates. Notably, outside of cluster 1, all isolates carried a nonsense mutation in *algB* (S199*), a positive regulator of *algD*, which is a GDP-mannose 6-dehydrogenase essential for alginate biosynthesis (40). In addition, a small group of isolates in cluster 5 encoded an additional nonsense mutation in AlgB (W191*), resulting in a second independent truncation event (Fig. 3A). Phenotypic assessment of isolates harboring *algU* or *algB* mutations in the fixed *mucA* loss-of-function background shows loss of mucoidy (Fig. 3E), indicating that a fixed *mucA* frameshift mutation initially predisposed the population to alginate production, while subsequent lineage-specific mutations in *algU* and *algB* drove repeated reversions to the non-mucoid phenotype (Fig. 3F).

#### Efflux pump mutations are not predictive of antibiotic resistance phenotypes

**Fig 4:**
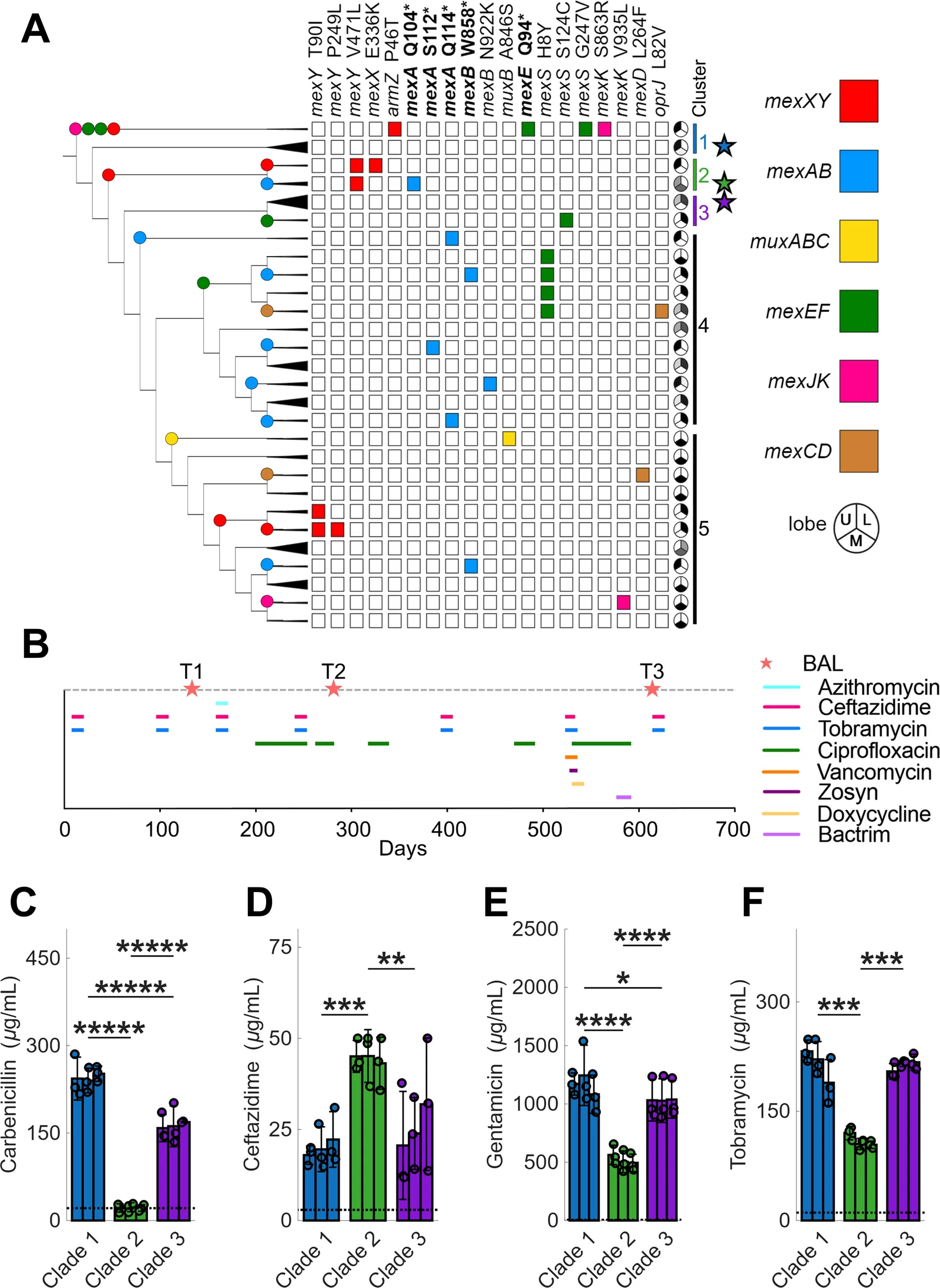
Efflux pumps are frequently mutated in CF isolates. (**A**) Mutations affecting efflux mechanisms are marked in the phylogeny tree of *P. aeruginosa* isolates. Adjacent strains with identical mutations were collapsed into the same leaf for clarity, with the size of the wedges representing the number of isolates collapsed into each leaf. Colored dots represent where each mutation likely occurred in the phylogeny tree. Bold *x*-axis labels represent nonsense mutations likely leading to loss of protein function. Cluster numbers are adjacent to the lobe pie graphs, and the stars denote which strains were chosen for the antibiotic resistance assays, shown in the subsequent panels. (**B**) Timeline of *P. aeruginosa* isolate collection and antibiotic usage over the study period. Peach stars denote the dates of bronchoalveolar lavage (BAL) sampling corresponding to time points T1, T2, and T3, and horizontal bars indicate periods of antibiotic administration. IC_50_ values were determined for isolates from three clusters for (**C**) carbenicillin, (**D**) ceftazidime, (**E**) gentamicin, and (**F**) tobramycin. These clusters are denoted by stars in panel A. For each cluster, three strains collected at time point 1 from the right upper lobe were analyzed. Each dotted line represents the average IC_50_ value for three replicates of PAO1. No statistically significant differences were found between strains within the same clusters. Asterisks denote significance levels for pairwise comparison between clusters: *****P < 10^−5^, ****P < 10^−4^, ***P < 10^−3^, **P < 10^−2^, and *P < 0.05.

Our analysis of unfixed mutations found a wealth of non-synonymous mutations in genes encoding multidrug efflux pumps implicated in antibiotic resistance. In many instances, the same genes acquired missense or nonsense mutations in multiple lineages from distinct clusters, suggesting parallel adaptation to antibiotic treatments (Fig. 4A).

We observed many mutations in *mex* genes encoding the multidrug efflux pumps that act as the *P. aeruginosa* main line of defense against antibiotics and other toxic compounds. We found three independent instances of mutations in *mexS,* a regulator of MexEF, which confers resistance to chloramphenicol, sulfamethoxazole, trimethoprim, and fluoroquinolones (41, 42). These mutations were present in isolates from the upper, middle, and lower lobes of the right lung, and some of the resultant amino acid changes were previously reported to increase antibiotic resistance (43). We also found missense mutations in *mexXY* in two distantly related clusters, present in all lobes. The MexXY transporter is the primary efflux mechanism for aminoglycosides such as tobramycin, which is a first-line treatment for *P. aeruginosa* CF infections (44). In both clusters, certain lineages acquired sequential point mutations in *mexXY* (*mexY* V471L and *mexX* E336K; *mexY* T90I and *mexY* P249L), suggesting adaptation of the efflux pump to specific drugs (Fig. 4A). Previous studies have shown that mutations in *mexXY* can enhance resistance to aminoglycosides (45), but potentially at the cost of increased susceptibility to other antibiotics (46). We also found seven independent mutation events in *mexAB*, the primary efflux mechanism for β-lactams such as ceftazidime (47), observed across all time points, lobes, and multiple clusters. Surprisingly, six of the seven mutations lead to truncations, likely resulting in loss of protein function. Although MexAB and MexXY share partially overlapping substrate specificities, *mexAB* loss-of-function mutations are thought to increase MexXY activity by reducing competition for their shared outer membrane protein, OprM (48, 49). However, the effects of *mexAB* disruption on aminoglycoside resistance remain unclear. One previous study reported that removal of *mexB* did not affect aminoglycoside resistance (50), whereas another study reported that aminoglycoside resistance was reduced in Δ*mexAB* mutants and restored by *oprM* overexpression (48). Overall, these results indicate a strong selective pressure on efflux mechanisms affecting antibiotic resistance in the lung environment.

To probe whether the evolved efflux pump alleles were likely to increase resistance to the antibiotics taken by the patient (47), we measured resistance to drug substrates of MexAB—carbenicillin and ceftazidime—and MexXY—gentamicin and tobramycin in select isolates with different alleles. We picked three isolates from each of neighboring clusters 1, 2, and 3 (Fig. 4A). All three isolates from cluster 2 had a nonsense mutation in *mexA* and a missense mutation in *mexY*, while the isolates from clusters 1 and 3 had no unfixed mutations in genes encoding multidrug efflux pumps. All nine isolates were collected from the right upper lobe at time point 1, following a course of ceftazidime and tobramycin treatment (Fig. 4B). For each isolate, we determined both the IC_50_, which we defined as the drug concentration required to reduce the growth rate of a log-phase liquid culture by half relative to the absence of drug (Fig. 4C through F), and the MIC, which we defined as the drug concentration required for the final OD of the culture to remain under 0.1 after 20 h (Fig. S3). For each analysis, we used a range of drug concentrations well above MIC and IC_50_ for *P. aeruginosa* strain PAO1 to allow the detection of differences in resistance even in isolates that may have multiple drug resistance mutations (Fig. S4 to S7). We observed significant differences in resistance between clusters, while strains within a cluster showed no statistically significant differences in resistance for any of the antibiotics tested.

For all four drugs, the IC_50_ values for cluster 2 strains, with mutations in *mexXY* and nonsense mutation in *mexA*, differed significantly from clusters 1 and 3, which were largely similar to each other (Fig. S3). Cluster 2 isolates also exhibited nearly a 10-fold decrease in carbenicillin resistance, likely due to the nonsense mutation in *mexA*. However, cluster 2 isolates showed slightly higher resistance to ceftazidime, which was administered as a treatment and is also a substrate of MexAB, suggesting evolution of resistance through an alternative mechanism. Cluster 2 strains displayed lower resistance to both aminoglycosides tested, compared to the other clusters. MIC values showed similar trends except for ceftazidime, where cluster 3 isolates had slightly higher resistance (Fig. S3).

Together, these findings indicate that isolates from the same infection have substantial variation in antibiotic resistance, even among isolates collected from the same lung lobe and time point. Of note, isolates in clusters 1, 2, and 3 exhibited tobramycin MIC values 62-, 45-, and 74-fold above the CLSI resistance breakpoint, respectively (51) (Fig. S3). However, it remains unclear how much of the antibiotic resistance can be explained by mutations in efflux pumps alone. While the mutation pattern in the isolates in cluster 2 would suggest increased resistance to aminoglycosides and decreased resistance to β-lactams, the resistance profiles we measured for these strains do not support these expectations. Collectively, these observations suggest that additional mechanisms beyond *mex* efflux pumps play a major role in shaping the resistance profiles of the CF isolates and that *mex* mutations may be under selection for other reasons such as improved host colonization (49).

## DISCUSSION

The goal of this study was to assess the genotypic diversity of a chronic infection population derived from a single strain type (Fig. 1A) in an individual with a mild-to-moderate CF lung disease within and across separate lobes of the right lung and over time. The infection population was caused by a strain belonging to MLST499 (Fig. S1). The dimension of time for our sample set provides important clues as to how the population structure shifted over time. Our phylogenetic analysis revealed groups of closely related isolates with good bootstrap support that were comprised entirely of isolates from a single lobe, supporting the compartmentalization hypothesis outlined in the study by Jorth et al. (23) but one cannot exclude the possibility that with the analysis of a larger number of strains, different lineages would be present in different regions of the lung. Our data set also shows that individual lobes contain multiple lineages and that specific lineages can be found across multiple lobes. In addition, while a particular genotype may dominate a particular lobe at a single time point, this same genotype may not persist in the same lobe or even in the overall population at subsequent time points (Fig. 1C).

Our analyses suggest that evolutionary rates were not uniform throughout the lung (Fig. 2A, cluster 1). These differences may indicate that local factors, such as inflammation and the concomitant production of microbe damaging products or damage which may increase access to nutrients that may alter growth rate or population size, may impose distinct evolutionary pressures. In addition, specific unfixed mutations may impact mutation rate. We did not observe mutations in genes for which loss of function leads to a higher mutation rate.

We found that at singular time points, particular genotypes may, indeed, have been isolated to a single lobe of the lung (Fig. 3B and C), and this finding in the context of mild-to-moderate disease resembles what has previously been described for more advanced lung disease settings in which there is compartmentalization (23, 27). However, when multiple time points were considered, broad population shifts and changes in the location of particular genotypes hint that strict compartmentalization is not faithfully maintained over time (Fig. 2A). Furthermore, migration rates were discontinuous in time with mixing occurring, especially between the upper and the lower lobe around T2, followed by differentiation across lobes being rebuilt. It is important to note that there were changes in antibiotics with the introduction of ciprofloxacin also around T2 (Fig. 4B), suggesting that changes in disease state may have contributed to increased rates of migration. Because these populations were neither static nor isolated, they do not support a model in which differences in environment within specific lobes (e.g., drug access or extent of damage) was a major driver of evolution in this patient.

Moreover, the *P. aeruginosa genes* mutated in this individual with CF are largely consistent with those reported in prior CF longitudinal studies. Notably, 7 of the 10 most frequently mutated genes identified across 14 studies of *in vivo* evolution in the CF lung were also observed here (*algU*, *mucA*, *lasR*, *mexA*, *mexB*, *migA*, and *pvdS*) (Table S2) (52). This concordance supports the well-established convergent evolutionary trajectories of *P. aeruginosa* in the CF lung.

Our analyses found evidence of parallel mutations in different clusters and recent migration of genotypes across different lobes leading to their co-existence (Fig. 3). This finding may explain the remarkable similarities in transcriptome profiles from different lobes despite genetic compartmentalization (53). One example was in the regulation of alginate production. While all isolates had identical loss-of-function mutations in *mucA,* which promotes mucoidy, we found multiple examples of mutations in *algU*, *algB*, and potentially other genes such as *algP* and *algL* that suppressed this phenotype (Fig. 3A and E). The extensive genome sequence data generated in this study provide a valuable resource for future investigations into additional mutations that may influence the fitness of strains with altered activity within the Muc–Alg regulatory pathway. For instance, cluster 1 strains with intact AlgB also carry different alleles of *migA*, which encodes a glycosyltransferase that modifies lipopolysaccharide (LPS), and PA4776, which encodes the sensor kinase PmrB, another regulator of LPS modification (Data set S1) (54, 55). Furthermore, we found a Q45* *lasR* allele fixed in the population that is predicted to lead to loss-of-function. The ubiquity of this allele is interesting, as *lasR* loss-of-function mutations have been associated with worse patient outcomes (56, 57). Despite this, the individual with CF studied here has maintained relatively strong pulmonary function for someone chronically colonized by *P. aeruginosa*. Together, these findings highlight that the clinical impact of specific bacterial genotypes cannot be inferred from single mutations alone and that disease severity likely reflects the combined effects of host factors, microbial community context, and the broader genetic background of the infecting population.

The results from our antibiotic resistance testing emphasize the need to culture many strains from an infection when determining an effective treatment protocol, as we demonstrate that different subpopulations cultured from the same environment have significantly different antibiotic resistance profiles (Fig. 4C through F). Apart from antibiotic resistance, mutations in efflux pumps affect other CF-relevant phenotypes, such as quorum sensing and virulence. For instance, loss of MexAB function, one of the most ubiquitous mutations in this study, can lead to overproduction and accumulation of quorum sensing molecules (49, 58), which has been suggested to increase tissue invasiveness in the CF lung (49). Similarly, *mexEF* overexpression has been reported to reduce virulence due to attenuated quorum sensing (59), and *mexXY* overexpression was shown to relieve oxidative stress. Indeed, mutations in multidrug resistance mechanisms often emerge even in patients that were never exposed to antibiotic treatments (60), underscoring that these systems play broader physiological roles beyond drug efflux alone. Together, these observations indicate that remodeling of efflux mechanisms is a central component of *P. aeruginosa* adaptation to the lung environment. As a result, mutations affecting antibiotic resistance may arise in response to diverse selective pressures unrelated to clinical antibiotic use, complicating efforts to predict resistance phenotypes from genotype alone. In further support of factors other than drug resistance contributing to Mex evolution was the absence of *mexZ* mutations which are commonly associated with drug resistance in CF (61).

Chronic *P. aeruginosa* infections in the CF lung exist as dynamic and genetically diverse populations rather than uniform monocultures. As our findings demonstrate, even within a single species, the relative success of individual lineages can shift across both space and time. This heterogeneity has important implications in the context of antibiotic resistance and personalized medicine. A clearer understanding of the complexity of chronic infection populations can inform more effective treatment strategies, emphasizing the need for clinical approaches that account for the presence of diverse and evolving bacterial communities rather than relying on the characterization of a single representative isolate.

## MATERIALS AND METHODS

### Sample collection from a human subject

Following written informed consent, the person with CF underwent flexible bronchoscopy. During the BAL procedures, the right lung was sampled in the order of upper lobe (RUL), middle lobe (RML), and then lower lobe (RLL). Following the procedure, the person with CF was monitored until they were stable for discharge. An aliquot of the BAL fluid from the RUL was sent to the DHMC clinical microbiology laboratory for routine culture analysis, and a separate aliquot was sent to our lab for isolate purification and banking as described below.

### Strains, media, and growth conditions

Strains were maintained in lysogeny broth (LB) medium, which consists of 10 g tryptone, 5 g NaCl, and 5 g yeast extract per liter distilled H_2_O or LB agar which also contains 15 g of Bacto-agar per liter. To examine colony morphologies, isolates were stamped from 96-well freezer stock plate onto *Pseudomonas* isolation agar (PIA) plates (Difco 292710) and incubated in a humidity chamber at 37°C for 48 h. All isolates, in liquid culture and on solid plates, were grown at 37°C.

### Isolation of colonies from bronchoalveolar lavage and expectorated sputum

Immediately after sample collection, BAL fluid at different dilutions in phosphate-buffered saline was spread directly onto PIA and blood agar plates with a metal spreading tool and incubated at 37°C for 48 h. Individual colonies were picked from plates with well-isolated colonies, patched onto PIA, and grown for 36–48 h. These colonies were then stamped into liquid LB in a 96-well plate via 48-pin replicator and allowed to grow for 48 h. Subsequently, 100 µL of this culture was combined with 100 µL of 50% glycerol stock and frozen for storage at −80°C.

### Specific PCR analysis and single gene sequencing

Epicentre Yeast DNA Extraction Kit was used for gDNA extraction, with omission of the RNase incubation step for select strains. For *lasR* and *mucA* amplification, Taq Phusion polymerase was used in a total reaction volume of 25 µL (17.5 µL H_2_O, 5 µL GC buffer, 1.5 µL DMSO, 0.5 µL of dNTPs, forward primer, reverse primer, and Taq Phusion, plus 1 µL gDNA per tube). Primers were as follows: LasR_seq_1, 5′ CAA ACG CTG CGG TCT ATT GTT AAG TG 3′; LasR_seq_2 (Jack Hammond), 5′ CAG TCG TTT CGA GAA TGG CGA GAA C 3′; mucA_forward_seq+amp_RVG, 5′ TTG AGT TAC GAA GAT ATC GCC ACC GTG 3′; and mucA_REVERSE_seq+amp_RVG, 5′ GCG CTC GTA GAC GAA GGT GCC 3′. The resulting PCR products were run on a 1% agarose gel with SYBR Green DNA visualization stain to ensure specific amplification. Subsequently, the PCR amplicon was purified with PCR Purification Kit by Qiagen and Sanger sequenced by the Dartmouth Molecular Biology Core Facility. The resulting sequences were aligned and analyzed with SnapGene against the PAO1 reference genome.

### Library preparation and sequencing

Strains were inoculated from the frozen plates into 96 well plates containing into 300 µL of LB and grown overnight at 37°C. Beckman GenFind V2 kits were used to extract genomic DNA, which was subsequently normalized using the Qubit dsDNA BR Assay kit. Nextera XT kits are used to construct libraries consisting of roughly 500 basepair DNA size selected fragments and were normalized with the Qubit dsDNA HS Assay kit. Bioanalyzer analysis was submitted to the Roy J. Carver Technology Center at the University of Illinois Urbana-Champaign. Libraries were sequenced with Illumina HiSeq2500 platform at the Roy J. Carver Technology Center to obtain 250 nucleotide paired end reads. An isolate was chosen for Nanopore sequencing at the same center to serve as a reference for intrapopulation mutation analyses.

### Reference genome assembly and multi-locus sequence type determination

Internal reference for isolate T1_RLL_B5 was assembled from Oxford Nanopore long reads and Illumina Hiseq2500 paired end reads for error correction under Unicycler was used under “—conservative” mode for long read assembly. Annotation for internal reference genome was performed with Contigs of non-reference genomes were built from HiSeq reads with SPAdes assembler to explore gene content (62). Settings for spades were performed under “—careful” settings with both forward and reverse fastq reads. Coverage of contigs ranged from 20× to 121× and N50 ranged from 2,731 to 58,765 bp within HiSeq2500 sequenced genomes. Annotation of Nanopore assemblies was performed with prokka (63) parameters “--genus *Pseudomonas* --usegenus --species *aeruginosa* –rfam” and a PAO1 protein reference database.

Multi-locus sequence type (MLST) was identified by aligning reads to the PubMLST database using the software package ARIBA (64). Multi-locus sequence types were identified using *de novo* contigs—assembled from Illumina short reads of T1_RLL_B5 using SPAdes software, --careful setting alignment—against the PAO1 reference genome using MUMmer software. T1_RLL_B5 sequences for the following MLST loci ("*acsA*" "*aroE*" "*guaA*" "*mutL*" "*nuoD*" "*ppsA*" "*trpE*") were extracted and queried into PUBMLST.org to determine the sequence type (ST). Loci sequences from the PAO1 reference were also submitted to validate workflow.

### Quality filtering and SNP analysis

After high-throughput sequencing of isolates, raw HiSeq reads were trimmed with trimmotatic with “2:36:10 LEADING:20 TRAILING:20 MAXINFO:50:0.97 MINLEN:50” settings (65) and aligned to the internal reference. With samtools and breseq (33, 66), SNPs and indels that varied across the population were identified. After initial phylogenetic analyses of the SNP calls by breseq revealed a phylogenetic tree with low boot strap values and the appearance of rampant recombination, we undertook an unbiased filtering of our data to remove genomes and positions that were aberrant due to low or high read depth or low consensus scores. Consensus scores for SNPs were calculated as the sum of PHRED quality scores of the bases which support a SNP divided by the sum of all quality scores of bases mapped at that position. The filtering was performed as follows. Step 1: isolates were removed if the average read depth was <30, as low read depth led to a high frequency of missed SNP calls (52 out of 527 isolate genomes removed). Step 2: for each genome, the number of positions with a consensus score of less than 0.9 was determined. Isolate genomes for which this value was more than 2 standard deviations higher than the average were removed (25 isolate genomes removed). Our analyses suggested samples with a high number of positions with a low consensus score likely contained contamination from another strain which impaired SNP calling even if present at a low level. Step 3: we removed positions that had an average read depth across isolates of >2 standard deviations from the mean across all genomes or a read depth less than ten (17 out of 721 positions removed). Step 4: for each position, the number of isolates with consensus score <0.9 was determined. Positions were removed if the number of isolates with a low consensus score at that position was more than two standard deviations away from the mean (1 additional position removed). We carried forward 450 isolate genomes and 663 variable positions for further analyses. The impacts of SNPs were determined using SNPeff (67). Code for consensus score determination is available at https://github.com/debhogan-208/Ritz_Clay_450_Pa_isolates.

### Main phylogenetic tree construction

For phylogenetic tree construction, nexus files were constructed using either MEGAX or Geneious Prime software after initial nucleotide alignment between sample core SNPs (68). PAUP* tree building software (69) was used to perform a heuristic parsimony search to find the best tree distance tree based on these core SNPs. Bootstrapping was performed with nreps=100 (“bootstrap nreps=100 search=heuristic/addseq=random nreps=10 swap=tbr hold=1;”). iTOL was used to visualize trees and add metainformation (70). To create trees from distant strains of *Pseudomonas*, a core genome was created with Spine against available assembled genomes using consensus alignments. MUMmer was used to align assemblies to a core genome sequence at default settings (71, 72). Delta-filter was applied to alignment to exclude SNPs found in repeats.

### Population level analysis

A series of R packages were used for population level analyses. Binary tables were used for mutations found between all samples within a subject containing lobe and date information. Data manipulation and import were performed with “dplyr” and “poppr” (73). Rarefaction curves were created with the “vegan” package. Fst and Gst analysis were performed with StaMPP (74).

### Phylogenetic analysis of *P. aeruginosa* migration patterns among lobes of the right lung

To examine the rate and heterogeneity of evolution among isolates, we performed a root-to-tip regression of genetic distance (Fig. 1C) against sample day (34). The slope of this line approximates the average rate of evolution under a strict molecular clock model (Fig. 2A). To quantify the significance of this estimate, we used a permutation test in which the correlation between divergence and time was compared to a null distribution of the same statistic calculated with lobe locations of each tip shuffled for 10,000 replicates (75). The *P* value was calculated as the proportion of replicates in the null distribution with a correlation greater than or equal to the observed correlation.

Time-resolved phylogenetic trees were used to infer migration patterns of *P. aeruginosa* among the upper, middle, and lower lobes of the right lung using samples from BAL at days 128, 279, and 613. Nucleotides at 663 variable sites among isolates were concatenated to form input sequences. Markov chain Monte Carlo was used to sample the posterior distribution of tree topologies, branch lengths, and node heights under a uncorrelated lognormally distributed relaxed molecular clock model implemented in BEAST 2.6.7 (35, 36). Nucleotide substitution probabilities were modeled using the HKY substitution model (76). A coalescent Bayesian skyline with five intervals was selected as the tree prior (77). Migration among lobes was modeled using a three state asymmetric continuous time Markov “mugration” model implemented in the BEAST classic package v1.6.0 (35, 37). MCMC was run for 1 × 10^9^ iterations, with a 10% burn-in. Convergence of all parameter estimates (excluding branch rate categories) was assessed as having effective sample size (ESS) values >200 using Tracer v1.7.3 (78). To reduce computational complexity, 9,000 trees were re-sampled from this distribution, and a maximum clade credibility tree was used to visualize the posterior distribution of time trees with sampled lobe locations at each node (Fig. 2B). To represent migration events among lobes, switches among lobes, in which a child node had a different location than its parent node, were quantified for each sampled tree (Fig. 2C). Tree analyses were performed with custom scripts written in R 4.5.1 (79), using the packages ape v5.8-1, treeio v1.34.0, ggtree v4.0.1, and qgraph v1.9.8 (80–83). Scripts and BEAST XML files to reproduce these analyses are available at https://github.com/debhogan-208/Ritz_Clay_450_Pa_isolates.

### Mucoid quantification

Each isolate was plated three times separately. Samples were diluted in PBS to yield between 30 and 150 colonies on PIA plates. The fraction of colonies with the mucoid morphology was assessed after 48 h. Before statistical comparisons, the assumption of normal distributions was checked using the Shapiro-Wilk test. The normal distribution assumption was rejected for at least one within-clusters data set for time point 3, but it was not rejected for the other time points. To account for this, the Wilcoxon rank-sum test was used to compare the groups in time point 3 (Fig. 3D), and one-way ANOVA was performed using the Tukey-Kramer correction to complete pairwise comparisons for the other time points (Fig. 3B and C).

### Antibiotic resistance assays

All resistance assay experiments were conducted in a modified M63 minimal medium (10 g/L (NH_4_)_2_SO_4_, 68 g/L KH_2_PO_4_, 2.5 mg/L FeSO_4_·7H_2_O) supplemented with 2 g/L glucose, 1 g/L casamino acids, 0.12 g/L MgSO_4_, and 0.5 mg/L thiamine. Drug stock solutions were freshly made from powder stocks (Sigma Aldrich and Cayman Chemical Company). We assessed the IC_50_ and MIC of three different CF isolates from three separate clusters, as well as the lab strains PA14 and PAO1, for a total of 11 strains. Each strain had three biological replicates, and each replicate was assayed on a separate day. Freshly grown colonies on LB plates were inoculated into LB media and grown overnight, diluted 200-fold in 96-well microtiter plates (Falcon) containing 200 μL of fresh medium and antibiotic solution per well. After inoculation, OD (absorbance at 595 nm) was recorded by a plate reader (Synergy Neo-2) every 5 min for at least 20 h. A concentration gradient of each antibiotic was set up over multiple 96-well plates. We used custom Matlab scripts to calculate the MIC, which we define as the drug concentration required to cause the final OD to be under 0.1 after 20 h, as well as the IC_50_, which we define as the drug concentration that causes the resulting steady-state growth rate of the population to be halved relative to the no-drug condition. We used fine gradients and interpolated between data points to find both resistance measurements. The IC_50_ is calculated by linear regression of log_2_(OD) during the exponential growth phase (Fig. S4 to S7). Any strains that did not cross the IC_50_ or MIC thresholds were assigned the highest drug concentration tested. Before statistical comparisons, the assumption of normal distributions was checked using the Shapiro-Wilk test. The normal distribution assumption was rejected for at least one within-clusters data set, but it was not rejected for the between-clusters data set. To account for this, Kruskal-Wallis was performed using the Bonferroni correction on strains within the same clusters, and one-way ANOVA was performed using the Tukey-Kramer correction on strains between clusters. When assessing statistical differences between the different clusters, antibiotic resistance measurements of biological replicates were first averaged together.

### Collapsed phylogenetic trees

The multidrug efflux pump and alginate collapsed phylogenetic trees were derived from the main phylogeny shown in Fig. 1B. Strains carrying unfixed non-synonymous mutations relative to the ancestor in the selected genes were identified and annotated with the strains’ lobe locations, cluster numbers, and the genes’ color categories. Adjacent strains sharing identical non-synonymous mutations were collapsed into the same leaf by pruning redundant branches. In the pruned trees, wedges at each leaf are proportional to the number of strains represented by that branch. In the multidrug efflux pump tree, stars denote which clusters were chosen to test in the antibiotic resistance assays. Transversion direction was determined by identifying the strand orientation of each gene of interest using online resources via pseudomonas.com (84). Code for construction of the trees is available at https://github.com/debhogan-208/Ritz_Clay_450_Pa_isolates.

### Grape tree

The grape tree was made from a minimum spanning network generated from the SNP alignment and visualized in GrapeTree version 1.5 (85).

## SUPPLEMENTAL MATERIAL

Data set S1 msystems.00238-26-s0001.xlsx Variable SNPs across the population.

Data set S2 msystems.00238-26-s0002.xlsx Variable indels across the population.

Supplemental material msystems.00238-26-s0003.pdf Supplemental figures and tables.

ASM does not own the copyrights to Supplemental Material that may be linked to, or accessed through, an article. The authors have granted ASM a non-exclusive, world-wide license to publish the Supplemental Material files. Please contact the corresponding author directly for reuse.

## References

[1] Emerson J, Rosenfeld M, McNamara S, Ramsey B, Gibson RL. 2002. *Pseudomonas aeruginosa* and other predictors of mortality and morbidity in young children with cystic fibrosis. Pediatr Pulmonol 34:91–100. doi:10.1002/ppul.10127 12112774

[2] Kosorok MR, Zeng L, West SE, Rock MJ, Splaingard ML, Laxova A, Green CG, Collins J, Farrell PM. 2001. Acceleration of lung disease in children with cystic fibrosis after *Pseudomonas aeruginosa* acquisition. Pediatr Pulmonol 32:277–287. doi:10.1002/ppul.2009.abs 11568988

[3] Winstanley C, O’Brien S, Brockhurst MA. 2016. *Pseudomonas aeruginosa* evolutionary adaptation and diversification in cystic fibrosis chronic lung infections. Trends Microbiol 24:327–337. doi:10.1016/j.tim.2016.01.008 PMC4854172 26946977

[4] D’Argenio DA, Wu M, Hoffman LR, Kulasekara HD, Déziel E, Smith EE, Nguyen H, Ernst RK, Larson Freeman TJ, Spencer DH, et al.. 2007. Growth phenotypes of *Pseudomonas aeruginosa* lasR mutants adapted to the airways of cystic fibrosis patients. Mol Microbiol 64:512–533. doi:10.1111/j.1365-2958.2007.05678.x PMC2742308 17493132

[5] Diaz Caballero J, Clark ST, Coburn B, Zhang Y, Wang PW, Donaldson SL, Tullis DE, Yau YCW, Waters VJ, Hwang DM, et al.. 2015. Selective sweeps and parallel pathoadaptation drive *Pseudomonas aeruginosa* evolution in the cystic fibrosis lung. mBio 6:e00981-15. doi:10.1128/mBio.00981-15 PMC4556809 26330513

[6] Smith EE, Buckley DG, Wu Z, Saenphimmachak C, Hoffman LR, D’Argenio DA, Miller SI, Ramsey BW, Speert DP, Moskowitz SM, et al.. 2006. Genetic adaptation by *Pseudomonas aeruginosa* to the airways of cystic fibrosis patients. Proc Natl Acad Sci USA 103:8487–8492. doi:10.1073/pnas.0602138103 PMC1482519 16687478

[7] Wilder CN, Allada G, Schuster M. 2009. Instantaneous within-patient diversity of *Pseudomonas aeruginosa* quorum-sensing populations from cystic fibrosis lung infections. Infect Immun 77:5631–5639. doi:10.1128/IAI.00755-09 PMC2786440 19805523

[8] Clay ME, Hammond JH, Zhong F, Chen X, Kowalski CH, Lee AJ, Porter MS, Hampton TH, Greene CS, Pletneva EV, et al.. 2020. *Pseudomonas aeruginosa* lasR mutant fitness in microoxia is supported by an Anr-regulated oxygen-binding hemerythrin. Proc Natl Acad Sci USA 117:3167–3173. doi:10.1073/pnas.1917576117 PMC7022198 31980538

[9] Jeske A, Arce-Rodriguez A, Thöming JG, Tomasch J, Häussler S. 2022. Evolution of biofilm-adapted gene expression profiles in *lasR*-deficient clinical *Pseudomonas aeruginosa* isolates. NPJ Biofilms Microbiomes 8:6. doi:10.1038/s41522-022-00268-1 PMC8844440 35165270

[10] LaFayette SL, Houle D, Beaudoin T, Wojewodka G, Radzioch D, Hoffman LR, Burns JL, Dandekar AA, Smalley NE, Chandler JR, et al.. 2015. Cystic fibrosis-adapted *Pseudomonas aeruginosa* quorum sensing lasR mutants cause hyperinflammatory responses. Sci Adv 1:e1500199. doi:10.1126/sciadv.1500199 PMC4597794 26457326

[11] Skopelja-Gardner S, Theprungsirikul J, Lewis KA, Hammond JH, Carlson KM, Hazlett HF, Nymon A, Nguyen D, Berwin BL, Hogan DA, et al.. 2019. Regulation of *Pseudomonas aeruginosa*-mediated neutrophil extracellular traps. Front Immunol 10:1670. doi:10.3389/fimmu.2019.01670 PMC6657737 31379861

[12] Casilag F, Lorenz A, Krueger J, Klawonn F, Weiss S, Häussler S. 2016. The LasB elastase of *Pseudomonas aeruginosa* Acts in concert with alkaline protease AprA to prevent flagellin-mediated immune recognition. Infect Immun 84:162–171. doi:10.1128/IAI.00939-15 PMC4693985 26502908

[13] Govan JR, Deretic V. 1996. Microbial pathogenesis in cystic fibrosis: mucoid *Pseudomonas aeruginosa* and *Burkholderia cepacia*. Microbiol Rev 60:539–574. doi:10.1128/mr.60.3.539-574.1996 PMC239456 8840786

[14] Henry RL, Mellis CM, Petrovic L. 1992. Mucoid *Pseudomonas aeruginosa* is a marker of poor survival in cystic fibrosis. Pediatr Pulmonol 12:158–161. doi:10.1002/ppul.1950120306 1641272

[15] Li Z, Kosorok MR, Farrell PM, Laxova A, West SEH, Green CG, Collins J, Rock MJ, Splaingard ML. 2005. Longitudinal development of mucoid *Pseudomonas aeruginosa* infection and lung disease progression in children with cystic fibrosis. JAMA 293:581–588. doi:10.1001/jama.293.5.581 15687313

[16] Pulcrano G, Iula DV, Raia V, Rossano F, Catania MR. 2012. Different mutations in mucA gene of *Pseudomonas aeruginosa* mucoid strains in cystic fibrosis patients and their effect on algU gene expression. New Microbiol 35:295–305. 22842599

[17] Schurr MJ, Yu H, Martinez-Salazar JM, Boucher JC, Deretic V. 1996. Control of AlgU, a member of the sigma E-like family of stress sigma factors, by the negative regulators MucA and MucB and *Pseudomonas aeruginosa* conversion to mucoidy in cystic fibrosis. J Bacteriol 178:4997–5004. doi:10.1128/jb.178.16.4997-5004.1996 PMC178285 8759866

[18] Prickett MH, Hauser AR, McColley SA, Cullina J, Potter E, Powers C, Jain M. 2017. Aminoglycoside resistance of *Pseudomonas aeruginosa* in cystic fibrosis results from convergent evolution in the mexZ gene. Thorax 72:40–47. doi:10.1136/thoraxjnl-2015-208027 PMC5497499 27325751

[19] Lieberman TD, Flett KB, Yelin I, Martin TR, McAdam AJ, Priebe GP, Kishony R. 2014. Genetic variation of a bacterial pathogen within individuals with cystic fibrosis provides a record of selective pressures. Nat Genet 46:82–87. doi:10.1038/ng.2848 PMC3979468 24316980

[20] Murante D, Demers EG, Kurbessoian T, Ruzic M, Ashare A, Stajich JE, Hogan DA. 2023. Mrs4 loss of function in fungi during adaptation to the cystic fibrosis lung. mBio 14:e01171-23. doi:10.1128/mbio.01171-23 PMC10470810 37432019

[21] Demers EG, Biermann AR, Masonjones S, Crocker AW, Ashare A, Stajich JE, Hogan DA. 2018. Evolution of drug resistance in an antifungal-naive chronic *Candida lusitaniae* infection. Proc Natl Acad Sci USA 115:12040–12045. doi:10.1073/pnas.1807698115 PMC6255150 30389707

[22] Demers EG, Stajich JE, Ashare A, Occhipinti P, Hogan DA. 2021. Balancing positive and negative selection: in vivo evolution of Candida lusitaniae MRR1. mBio 12:e03328-20. doi:10.1128/mBio.03328-20 PMC8092287 33785623

[23] Jorth P, Staudinger BJ, Wu X, Hisert KB, Hayden H, Garudathri J, Harding CL, Radey MC, Rezayat A, Bautista G, et al.. 2015. Regional isolation drives bacterial diversification within cystic fibrosis lungs. Cell Host Microbe 18:307–319. doi:10.1016/j.chom.2015.07.006 PMC4589543 26299432

[24] Chung JC, Becq J, Fraser L, Schulz-Trieglaff O, Bond NJ, Foweraker J, Bruce KD, Smith GP, Welch M. 2012. Genomic variation among contemporary *Pseudomonas aeruginosa* isolates from chronically infected cystic fibrosis patients. J Bacteriol 194:4857–4866. doi:10.1128/JB.01050-12 PMC3430303 22753054

[25] Clark ST, Diaz Caballero J, Cheang M, Coburn B, Wang PW, Donaldson SL, Zhang Y, Liu M, Keshavjee S, Yau YCW, et al.. 2015. Phenotypic diversity within a *Pseudomonas aeruginosa* population infecting an adult with cystic fibrosis. Sci Rep 5:10932. doi:10.1038/srep10932 PMC4456944 26047320

[26] Fothergill JL, Mowat E, Ledson MJ, Walshaw MJ, Winstanley C. 2010. Fluctuations in phenotypes and genotypes within populations of *Pseudomonas aeruginosa* in the cystic fibrosis lung during pulmonary exacerbations. J Med Microbiol 59:472–481. doi:10.1099/jmm.0.015875-0 20019149

[27] Markussen T, Marvig RL, Gomez-Lozano M, Aanaes K, Burleigh AE, Hoiby N, Johansen HK, Molin S, Jelsbak L. 2014. Environmental heterogeneity drives within-host diversification and evolution of *Pseudomonas aeruginosa*. mBio 5:e01592-14. doi:10.1128/mBio.01592-14 PMC4172072 25227464

[28] Mowat E, Paterson S, Fothergill JL, Wright EA, Ledson MJ, Walshaw MJ, Brockhurst MA, Winstanley C. 2011. *Pseudomonas aeruginosa* population diversity and turnover in cystic fibrosis chronic infections. Am J Respir Crit Care Med 183:1674–1679. doi:10.1164/rccm.201009-1430OC 21297072

[29] Williams D, Evans B, Haldenby S, Walshaw MJ, Brockhurst MA, Winstanley C, Paterson S. 2015. Divergent, coexisting *Pseudomonas aeruginosa* lineages in chronic cystic fibrosis lung infections. Am J Respir Crit Care Med 191:775–785. doi:10.1164/rccm.201409-1646OC PMC4407486 25590983

[30] Workentine ML, Sibley CD, Glezerson B, Purighalla S, Norgaard-Gron JC, Parkins MD, Rabin HR, Surette MG. 2013. Phenotypic heterogeneity of *Pseudomonas aeruginosa* populations in a cystic fibrosis patient. PLoS One 8:e60225. doi:10.1371/journal.pone.0060225 PMC3616088 23573242

[31] Marvig Rasmus Lykke, Damkiær S, Khademi SMH, Markussen TM, Molin S, Jelsbak L. 2014. Within-host evolution of *Pseudomonas aeruginosa* reveals adaptation toward iron acquisition from hemoglobin. mBio 5:e00966-14. doi:10.1128/mBio.00966-14 PMC4010824 24803516

[32] Marvig R.L, Sommer LM, Molin S, Johansen HK. 2015. Convergent evolution and adaptation of *Pseudomonas aeruginosa* within patients with cystic fibrosis. Nat Genet 47:57–64. doi:10.1038/ng.3148 25401299

[33] Barrick JE, Colburn G, Deatherage DE, Traverse CC, Strand MD, Borges JJ, Knoester DB, Reba A, Meyer AG. 2014. Identifying structural variation in haploid microbial genomes from short-read resequencing data using breseq. BMC Genomics 15:1039. doi:10.1186/1471-2164-15-1039 PMC4300727 25432719

[34] Rambaut A, Lam TT, Max Carvalho L, Pybus OG. 2016. Exploring the temporal structure of heterochronous sequences using TempEst (formerly Path-O-Gen). Virus Evol 2:vew007. doi:10.1093/ve/vew007 PMC4989882 27774300

[35] Bouckaert R, Vaughan TG, Barido-Sottani J, Duchene S, Fourment M, Gavryushkina A, Heled J, Jones G, Kuhnert D, Maio N, et al.. 2019. BEAST 2.5: an advanced software platform for bayesian evolutionary analysis. PLoS Comput Biol 15:e1006650. doi:10.1371/journal.pcbi.1006650 PMC6472827 30958812

[36] Drummond AJ, Ho SYW, Phillips MJ, Rambaut A. 2006. Relaxed phylogenetics and dating with confidence. PLoS Biol 4:e88. doi:10.1371/journal.pbio.0040088 PMC1395354 16683862

[37] Lemey P, Rambaut A, Drummond AJ, Suchard MA. 2009. Bayesian phylogeography finds its roots. PLoS Comput Biol 5:e1000520. doi:10.1371/journal.pcbi.1000520 PMC2740835 19779555

[38] Martin DW, Schurr MJ, Mudd MH, Govan JR, Holloway BW, Deretic V. 1993. Mechanism of conversion to mucoidy in *Pseudomonas aeruginosa* infecting cystic fibrosis patients. Proc Natl Acad Sci USA 90:8377–8381. doi:10.1073/pnas.90.18.8377 PMC47359 8378309

[39] Schurr MJ, Martin DW, Mudd MH, Deretic V. 1994. Gene cluster controlling conversion to alginate-overproducing phenotype in *Pseudomonas aeruginosa*: functional analysis in a heterologous host and role in the instability of mucoidy. J Bacteriol 176:3375–3382. doi:10.1128/jb.176.11.3375-3382.1994 PMC205510 8195094

[40] Leech AJ, Sprinkle A, Wood L, Wozniak DJ, Ohman DE. 2008. The NtrC family regulator AlgB, which controls alginate biosynthesis in mucoid *Pseudomonas aeruginosa*, binds directly to the algD promoter. J Bacteriol 190:581–589. doi:10.1128/JB.01307-07 PMC2223705 17981963

[41] Köhler T, Michéa-Hamzehpour M, Henze U, Gotoh N, Curty LK, Pechère JC. 1997. Characterization of MexE-MexF-OprN, a positively regulated multidrug efflux system of *Pseudomonas aeruginosa*. Mol Microbiol 23:345–354. doi:10.1046/j.1365-2958.1997.2281594.x 9044268

[42] Mazza L, Bory A, Luscher A, Kloehn J, Wolfender J-L, van Delden C, Köhler T. 2024. Multidrug efflux pumps of *Pseudomonas aeruginosa* show selectivity for their natural substrates. Front Microbiol 15:1512472. doi:10.3389/fmicb.2024.1512472 PMC11754269 39850140

[43] Sobel ML, Neshat S, Poole K. 2005. Mutations in PA2491 (mexS) promote MexT-dependent mexEF-oprN expression and multidrug resistance in a clinical strain of *Pseudomonas aeruginosa*. J Bacteriol 187:1246–1253. doi:10.1128/JB.187.4.1246-1253.2005 PMC545639 15687188

[44] Morita Y, Tomida J, Kawamura Y. 2012. MexXY multidrug efflux system of *Pseudomonas aeruginosa*. Front Microbiol 3:408. doi:10.3389/fmicb.2012.00408 PMC3516279 23233851

[45] Lau CF, Hughes D, Poole K. 2014. MexY-promoted aminoglycoside resistance in *Pseudomonas aeruginosa*: involvement of a putative proximal binding pocket in aminoglycoside recognition. mBio 5:e01068-14. doi:10.1128/mBio.01068-14 PMC3994515 24757215

[46] Vettoretti L, Plésiat P, Muller C, El Garch F, Phan G, Attrée I, Ducruix A, Llanes C. 2009. Efflux unbalance in *Pseudomonas aeruginosa* isolates from cystic fibrosis patients. Antimicrob Agents Chemother 53:1987–1997. doi:10.1128/AAC.01024-08 PMC2681539 19258280

[47] Masuda N, Sakagawa E, Ohya S, Gotoh N, Tsujimoto H, Nishino T. 2000. Substrate specificities of MexAB-OprM, MexCD-OprJ, and MexXY-oprM efflux pumps in *Pseudomonas aeruginosa*. Antimicrob Agents Chemother 44:3322–3327. doi:10.1128/AAC.44.12.3322-3327.2000 PMC90200 11083635

[48] Seupt A, Schniederjans M, Tomasch J, Haussler S. 2020. Expression of the MexXY aminoglycoside efflux pump and presence of an aminoglycoside-modifying enzyme in clInical *Pseudomonas aeruginosa* isolates are highly correlated. Antimicrob Agents Chemother 65:e01166-20. doi:10.1128/AAC.01166-20 PMC7927871 33046496

[49] Laborda P, Lolle S, Hernando-Amado S, Alcalde-Rico M, Aanæs K, Martínez JL, Molin S, Johansen HK. 2024. Mutations in the efflux pump regulator MexZ shift tissue colonization by *Pseudomonas aeruginosa* to a state of antibiotic tolerance. Nat Commun 15:2584. doi:10.1038/s41467-024-46938-w PMC10959964 38519499

[50] Fruci M, Poole K. 2018. Aminoglycoside-inducible expression of the mexAB-oprM multidrug efflux operon in *Pseudomonas aeruginosa*: involvement of the envelope stress-responsive AmgRS two-component system. PLoS One 13:e0205036. doi:10.1371/journal.pone.0205036 PMC6173428 30289929

[51] Schuetz AN, Ferrell A, Hindler JA, Humphries R, Bobenchik AM. 2025. Overview of changes in the clinical and laboratory standards institute performance standards for antimicrobial susceptibility testing: M100 32nd and 33rd editions. J Clin Microbiol 63:e01623-23. doi:10.1128/jcm.01623-23 PMC12421849 40772786

[52] Camus L, Vandenesch F, Moreau K. 2021. From genotype to phenotype: adaptations of *Pseudomonas aeruginosa* to the cystic fibrosis environment. Microb Genom 7:mgen000513. doi:10.1099/mgen.0.000513 PMC8190622 33529147

[53] Kordes A, Preusse M, Willger SD, Braubach P, Jonigk D, Haverich A, Warnecke G, Häussler S. 2019. Genetically diverse *Pseudomonas aeruginosa* populations display similar transcriptomic profiles in a cystic fibrosis explanted lung. Nat Commun 10:3397. doi:10.1038/s41467-019-11414-3 PMC6667473 31363089

[54] Poon KKH, Westman EL, Vinogradov E, Jin S, Lam JS. 2008. Functional characterization of MigA and WapR: putative rhamnosyltransferases involved in outer core oligosaccharide biosynthesis of *Pseudomonas aeruginosa*. J Bacteriol 190:1857–1865. doi:10.1128/JB.01546-07 PMC2258888 18178733

[55] Moskowitz SM, Brannon MK, Dasgupta N, Pier M, Sgambati N, Miller AK, Selgrade SE, Miller SI, Denton M, Conway SP, et al.. 2012. PmrB mutations promote polymyxin resistance of *Pseudomonas aeruginosa* isolated from colistin-treated cystic fibrosis patients. Antimicrob Agents Chemother 56:1019–1030. doi:10.1128/AAC.05829-11 PMC3264203 22106224

[56] Hammond JH, Dolben EF, Smith TJ, Bhuju S, Hogan DA. 2015. Links between Anr and quorum sensing in *Pseudomonas aeruginosa* biofilms. J Bacteriol 197:2810–2820. doi:10.1128/JB.00182-15 PMC4524035 26078448

[57] Hoffman LR, Kulasekara HD, Emerson J, Houston LS, Burns JL, Ramsey BW, Miller SI. 2009. *Pseudomonas aeruginosa lasR* mutants are associated with cystic fibrosis lung disease progression. J Cyst Fibros 8:66–70. doi:10.1016/j.jcf.2008.09.006 PMC2631641 18974024

[58] Alcalde-Rico M, Olivares-Pacheco J, Halliday N, Cámara M, Martínez JL. 2020. The impaired quorum sensing response of *Pseudomonas aeruginosa* MexAB-OprM efflux pump overexpressing mutants is not due to non-physiological efflux of 3-oxo-C12-HSL. Environ Microbiol 22:5167–5188. doi:10.1111/1462-2920.15177 32715566

[59] Kristensen R, Andersen JB, Rybtke M, Jansen CU, Fritz BG, Kiilerich RO, Uhd J, Bjarnsholt T, Qvortrup K, Tolker-Nielsen T, et al.. 2024. Inhibition of *Pseudomonas aeruginosa* quorum sensing by chemical induction of the MexEF-oprN efflux pump. Antimicrob Agents Chemother 68:e01387-23. doi:10.1128/aac.01387-23 PMC10848761 38189278

[60] Fraud S, Poole K. 2011. Oxidative stress induction of the MexXY multidrug efflux genes and promotion of aminoglycoside resistance development in *Pseudomonas aeruginosa*. Antimicrob Agents Chemother 55:1068–1074. doi:10.1128/AAC.01495-10 PMC3067074 21173187

[61] Frimodt-Moller J, Rossi E, Haagensen JAJ, Falcone M, Molin S, Johansen HK. 2018. Mutations causing low level antibiotic resistance ensure bacterial survival in antibiotic-treated hosts. Sci Rep 8:12512. doi:10.1038/s41598-018-30972-y PMC6104031 30131514

[62] Bankevich A, Nurk S, Antipov D, Gurevich AA, Dvorkin M, Kulikov AS, Lesin VM, Nikolenko SI, Pham S, Prjibelski AD, et al.. 2012. SPAdes: a new genome assembly algorithm and its applications to single-cell sequencing. J Comput Biol 19:455–477. doi:10.1089/cmb.2012.0021 PMC3342519 22506599

[63] Seemann T. 2014. Prokka: rapid prokaryotic genome annotation. Bioinformatics 30:2068–2069. doi:10.1093/bioinformatics/btu153 24642063

[64] Hunt M, Mather AE, Sánchez-Busó L, Page AJ, Parkhill J, Keane JA, Harris SR. 2017. ARIBA: rapid antimicrobial resistance genotyping directly from sequencing reads. Microb Genom 3:e000131. doi:10.1099/mgen.0.000131 PMC5695208 29177089

[65] Bolger AM, Lohse M, Usadel B. 2014. Trimmomatic: a flexible trimmer for illumina sequence data. Bioinformatics 30:2114–2120. doi:10.1093/bioinformatics/btu170 PMC4103590 24695404

[66] Li H, Handsaker B, Wysoker A, Fennell T, Ruan J, Homer N, Marth G, Abecasis G, Durbin R, Genome Project Data Processing S. 2009. The sequence alignment/map format and SAMtools. Bioinformatics 25:2078–2079. doi:10.1093/bioinformatics/btp352 PMC2723002 19505943

[67] Cingolani P, Platts A, Wang LL, Coon M, Nguyen T, Wang L, Land SJ, Lu X, Ruden DM. 2012. A program for annotating and predicting the effects of single nucleotide polymorphisms, SnpEff: SNPs in the genome of *Drosophila melanogaster* strain w1118; iso-2; iso-3. Fly (Austin) 6:80–92. doi:10.4161/fly.19695 PMC3679285 22728672

[68] Kumar S, Tamura K, Nei M. 1994. MEGA: molecular evolutionary genetics analysis software for microcomputers. Bioinformatics 10:189–191. doi:10.1093/bioinformatics/10.2.189 8019868

[6] Wilgenbusch JC, Swofford D. 2003. Inferring evolutionary trees with PAUP*. Curr Protoc Bioinform 00:6.4.1–6.4.28. doi:10.1002/0471250953.bi0604s00 18428704

[70] Letunic I, Bork P. 2019. Interactive tree of life (iTOL) v4: recent updates and new developments. Nucleic Acids Res 47:W256–W259. doi:10.1093/nar/gkz239 PMC6602468 30931475

[71] Ozer EA, Allen JP, Hauser AR. 2014. Characterization of the core and accessory genomes of *Pseudomonas aeruginosa* using bioinformatic tools spine and AGEnt. BMC Genomics 15:737. doi:10.1186/1471-2164-15-737 PMC4155085 25168460

[72] Kurtz S, Phillippy A, Delcher AL, Smoot M, Shumway M, Antonescu C, Salzberg SL. 2004. Versatile and open software for comparing large genomes. Genome Biol 5:R12. doi:10.1186/gb-2004-5-2-r12 PMC395750 14759262

[73] Kamvar ZN, Tabima JF, Grünwald NJ. 2014. Poppr: an R package for genetic analysis of populations with clonal, partially clonal, and/or sexual reproduction. PeerJ 2:e281. doi:10.7717/peerj.281 PMC3961149 24688859

[74] Pembleton LW, Cogan NOI, Forster JW. 2013. StAMPP: an R package for calculation of genetic differentiation and structure of mixed-ploidy level populations. Mol Ecol Resour 13:946–952. doi:10.1111/1755-0998.12129 23738873

[75] Duchêne S, Duchêne D, Holmes EC, Ho SYW. 2015. The performance of the date-randomization test in phylogenetic analyses of time-structured virus data. Mol Biol Evol 32:1895–1906. doi:10.1093/molbev/msv056 25771196

[76] Hasegawa M, Kishino H, Yano T. 1985. Dating of the human-ape splitting by a molecular clock of mitochondrial DNA. J Mol Evol 22:160–174. doi:10.1007/BF02101694 3934395

[77] Drummond AJ, Rambaut A, Shapiro B, Pybus OG. 2005. Bayesian coalescent inference of past population dynamics from molecular sequences. Mol Biol Evol 22:1185–1192. doi:10.1093/molbev/msi103 15703244

[78] Rambaut A, Drummond AJ, Xie D, Baele G, Suchard MA. 2018. Posterior summarization in bayesian phylogenetics using tracer 1.7. Syst Biol 67:901–904. doi:10.1093/sysbio/syy032 PMC6101584 29718447

[79] Team RC. 2017. R: a language and environment for statistical computing. https://www.r-project.org/.

[80] Wang LG, Lam TY, Xu S, Dai Z, Zhou L, Feng T, Guo P, Dunn CW, Jones BR, Bradley T, et al.. 2020. Treeio: an R package for phylogenetic tree input and output with richly annotated and associated data. Mol Biol Evol 37:599–603. doi:10.1093/molbev/msz240 PMC6993851 31633786

[81] Xu S, Li L, Luo X, Chen M, Tang W, Zhan L, Dai Z, Lam TT, Guan Y, Yu G. 2022. *Ggtree*: a serialized data object for visualization of a phylogenetic tree and annotation data. Imeta 1:e56. doi:10.1002/imt2.56 PMC10989815 38867905

[82] Epskamp S, Cramer AOJ, Waldorp LJ, Schmittmann VD, Borsboom D. 2012. qgraph: network visualizations of relationships in psychometric data. J Stat Soft 48:18. doi:10.18637/jss.v048.i04

[83] Paradis E, Claude J, Strimmer K. 2004. APE: analyses of phylogenetics and evolution in R language. Bioinformatics 20:289–290. doi:10.1093/bioinformatics/btg412 14734327

[84] Winsor GL, Van Rossum T, Lo R, Khaira B, Whiteside MD, Hancock REW, Brinkman FSL. 2009. *Pseudomonas* genome database: facilitating user-friendly, comprehensive comparisons of microbial genomes. Nucleic Acids Res 37:D483–8. doi:10.1093/nar/gkn861 PMC2686508 18978025

[85] Zhou Z, Alikhan N-F, Sergeant MJ, Luhmann N, Vaz C, Francisco AP, Carriço JA, Achtman M. 2018. GrapeTree: visualization of core genomic relationships among 100,000 bacterial pathogens. Genome Res 28:1395–1404. doi:10.1101/gr.232397.117 PMC6120633 30049790

