# Supplementary material for: Within-host population structure, migration, and parallel adaptive evolution of Pseudomonas aeruginosa in cystic fibrosis lung disease
Source: mSystems. 2026 Aug 25;11(9):e00238-26. doi: 10.1128/msystems.00238-26 (PMC13595944; doi:10.1128/msystems.00238-26)
Supplement: Supplementary file 1 [file msystems.00238-26-s0003.pdf]

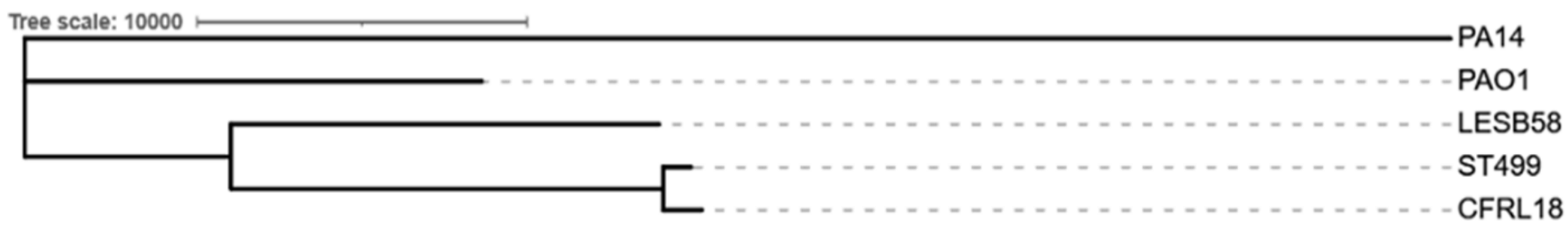

Figure S1: *P. aeruginosa* ST499 strain is more closely related to CFL18 than lab strains. Phylogenetic relationship between the internal reference strain (CFL18) relative to the MLST-matched outgroup (group ST499, strain ERR364087), common laboratory strains (PAO1 and PA14), and the Liverpool Epidemic strain LESB58. Bootstrap support values were 100 for every node.

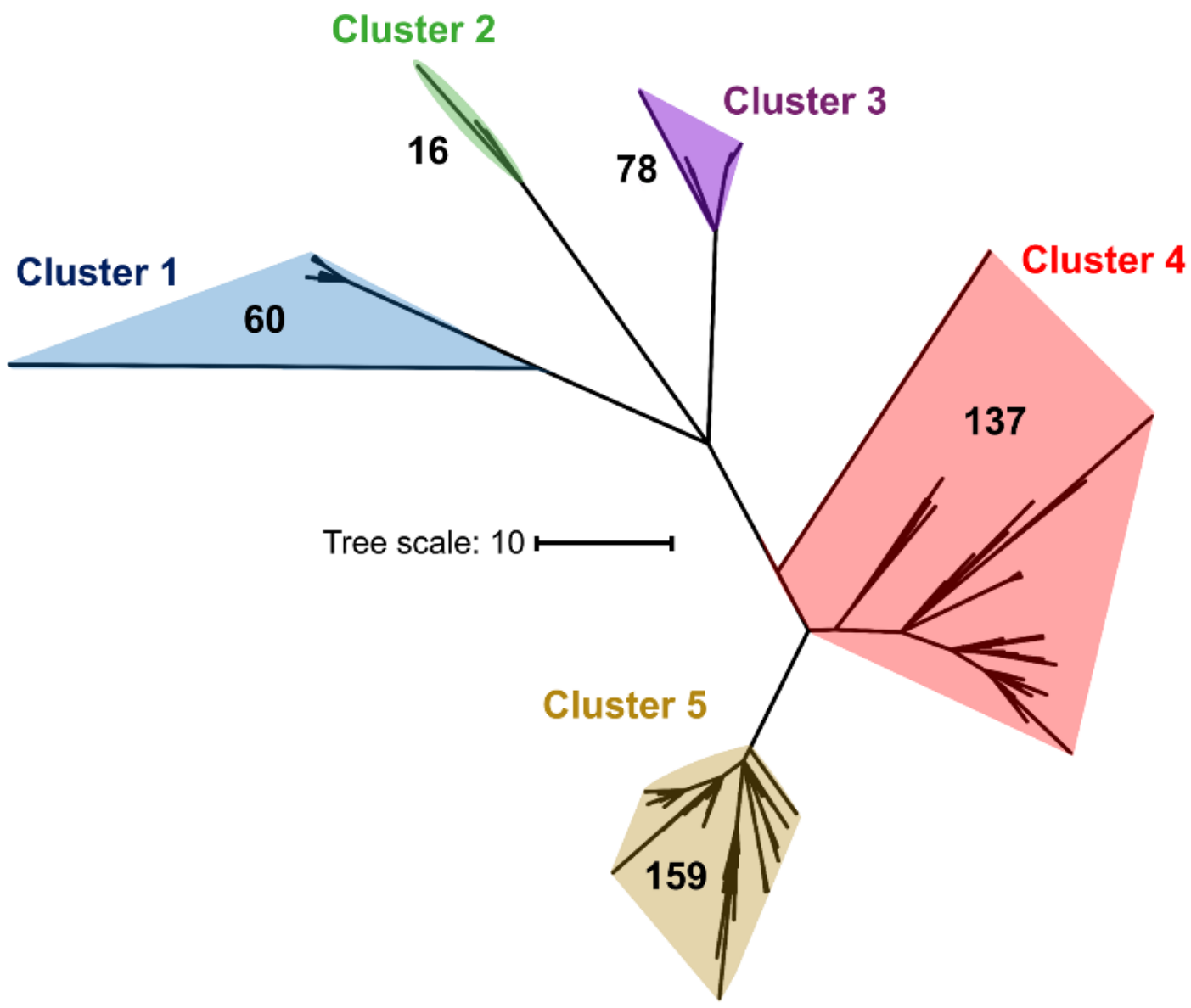

Figure S2: *P. aeruginosa* isolates form distinct clusters. Unrooted tree constructed from **Fig. 1C**. The colors denote the different clusters. The branch length corresponds to the number of detected SNPs.

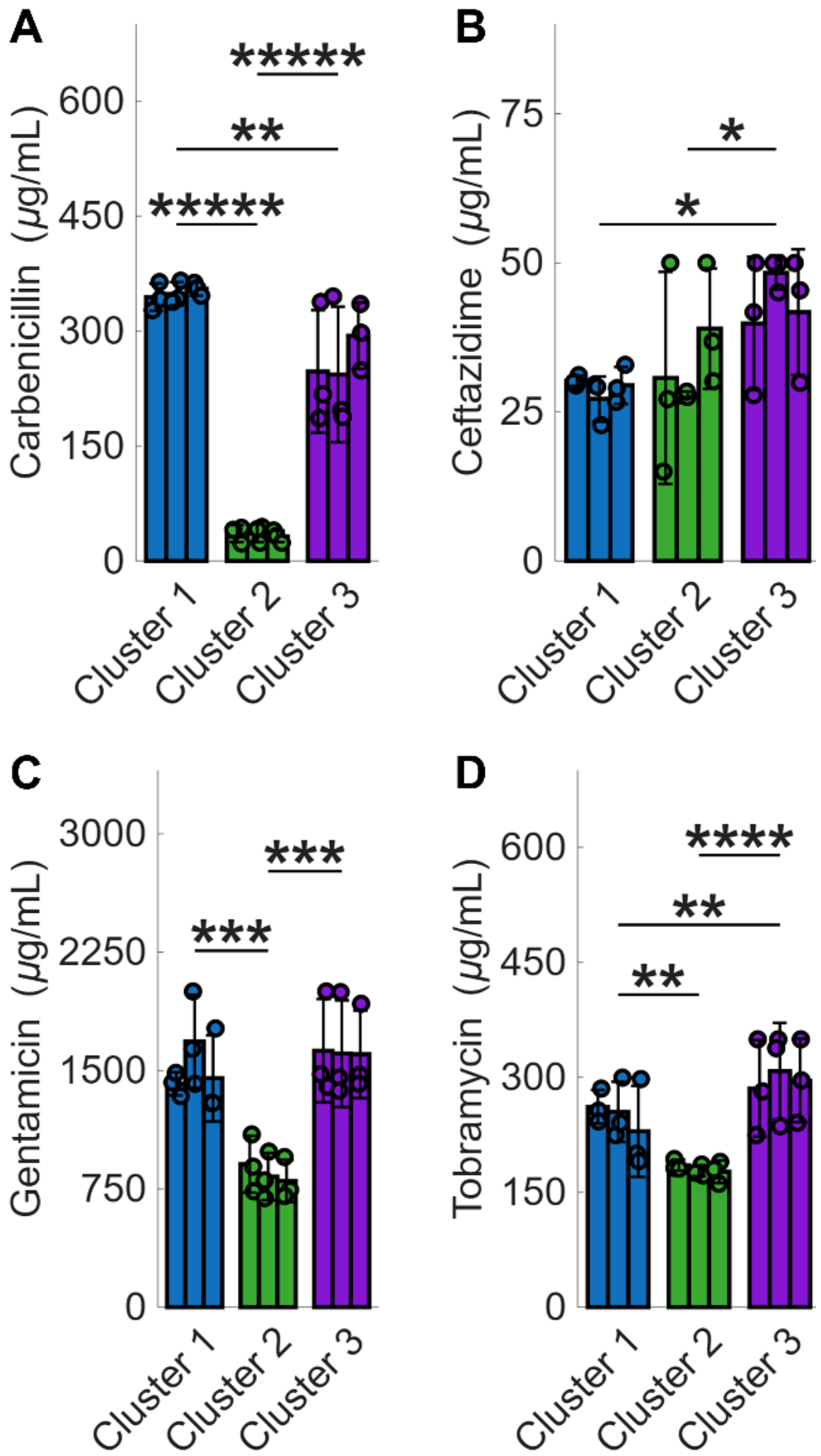

Figure S3: *P. aeruginosa* isolates have heterogeneous antibiotic MIC values. MIC values were determined for isolates from three clusters for **(A)** carbenicillin, **(B)** ceftazidime, **(C)** gentamicin, and **(D)** tobramycin. These clusters are denoted by stars in **Fig. 4A**. For each cluster, three strains collected at Timepoint 1 from the right upper lobe were analyzed. We calculate MIC as the drug concentration that causes the final OD of the culture to be under 0.1. No statistically significant differences were found between strains within the same clusters. Asterisks denote significance levels for pairwise comparison between clusters: ***** $p < 10^{-5}$, **** $p < 10^{-4}$, *** $p < 10^{-3}$, ** $p < 10^{-2}$, and * $p < 0.05$.

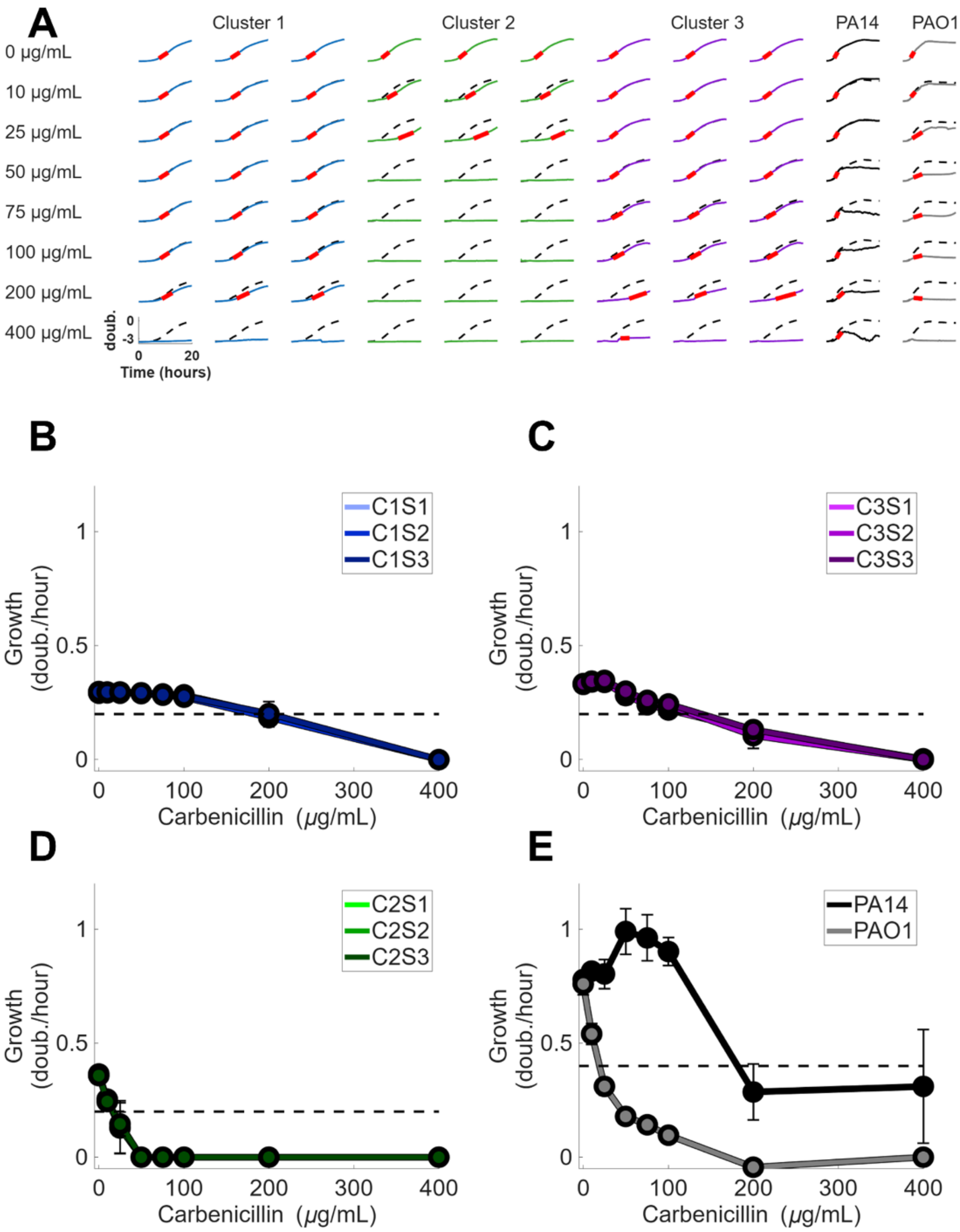

Figure S4: Calculation of carbenicillin resistances from cell growth during a drug exposure. (A) Isolates from clusters 1-3, PA14, and PAO1 following exposure to a gradient of carbenicillin, performed in three replicates. One representative plate is shown. Drug is added before the inoculation with cells at time = 0. The red line shows the steady-state growth following population recovery. The overlaid black dotted line represents growth in the absence of drug, which is the first row of each column. These growth curves were used to calculate the IC_{50} in **Fig. 4C** and MIC in **Fig S3A**. **(B-E)** Steady-state growth of each strain ("S") as a function of the carbenicillin dose for **(D)** Cluster 1, **(E)** Cluster 2, **(F)** Cluster 3, and **(G)** lab strains. The horizontal line indicates half of the growth rate during exponential growth in the absence of drug. We calculate IC_{50} as the drug concentration that halves the maximum growth rate reached after exposure.

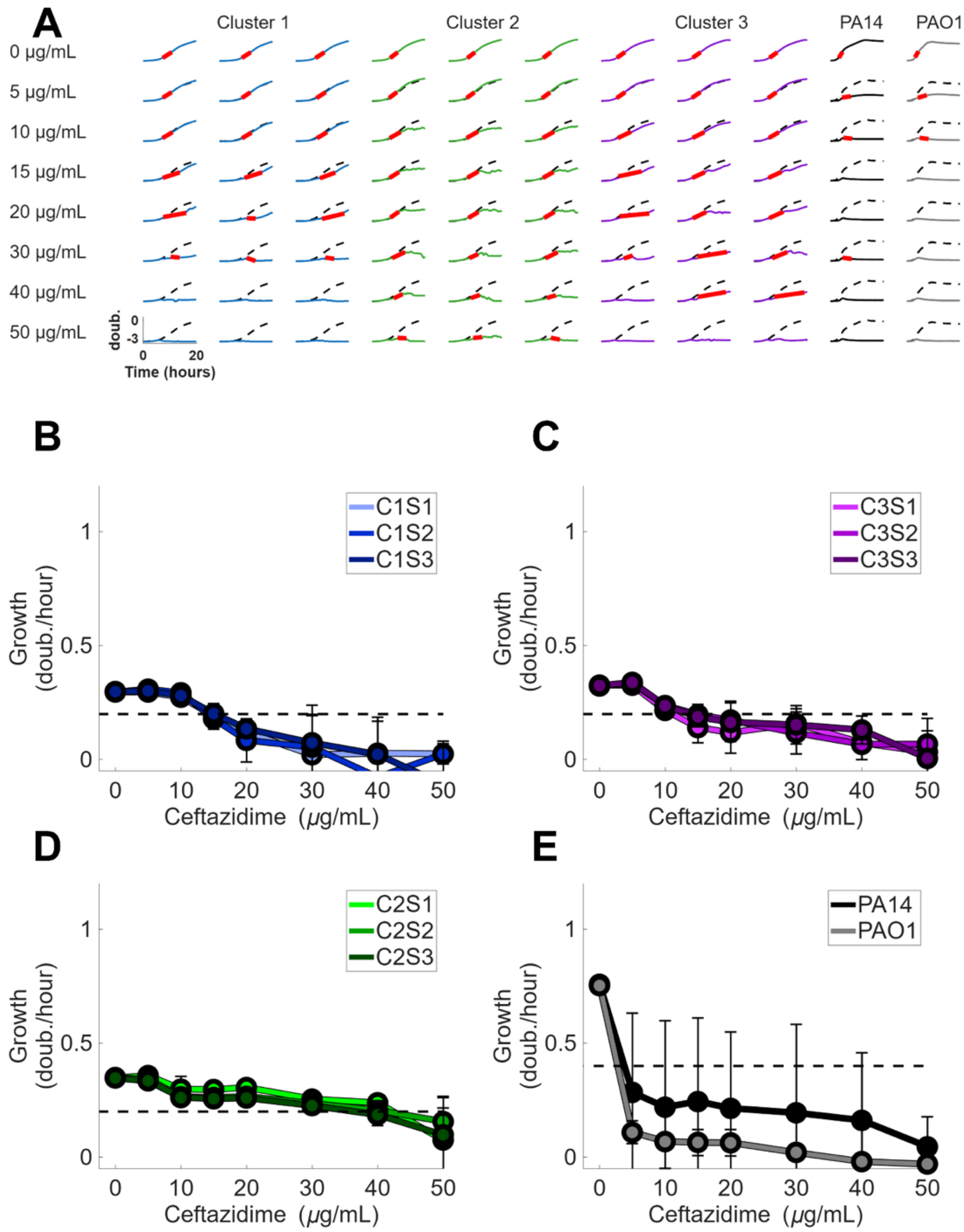

Figure S5: Calculation of ceftazidime resistances from cell growth during a drug exposure. (A) Isolates from clusters 1-3, PA14, and PAO1 following exposure to a gradient of ceftazidime, performed in three replicates. One representative plate is shown. Drug is added before the inoculation with cells at time = 0. The red line shows the steady-state growth following population recovery. The overlaid black dotted line represents growth in the absence of drug, which is the first row of each column. These growth curves were used to calculate the IC_{50} in **Fig. 4D** and MIC in **Fig S3B**. **(B-E)** Steady-state growth of each strain ("S") as a function of the ceftazidime dose for **(D)** Cluster 1, **(E)** Cluster 2, **(F)** Cluster 3, and **(G)** lab strains. The horizontal line indicates half of the growth rate during exponential growth in the absence of drug. We calculate IC_{50} as the drug concentration that halves the maximum growth rate reached after exposure.

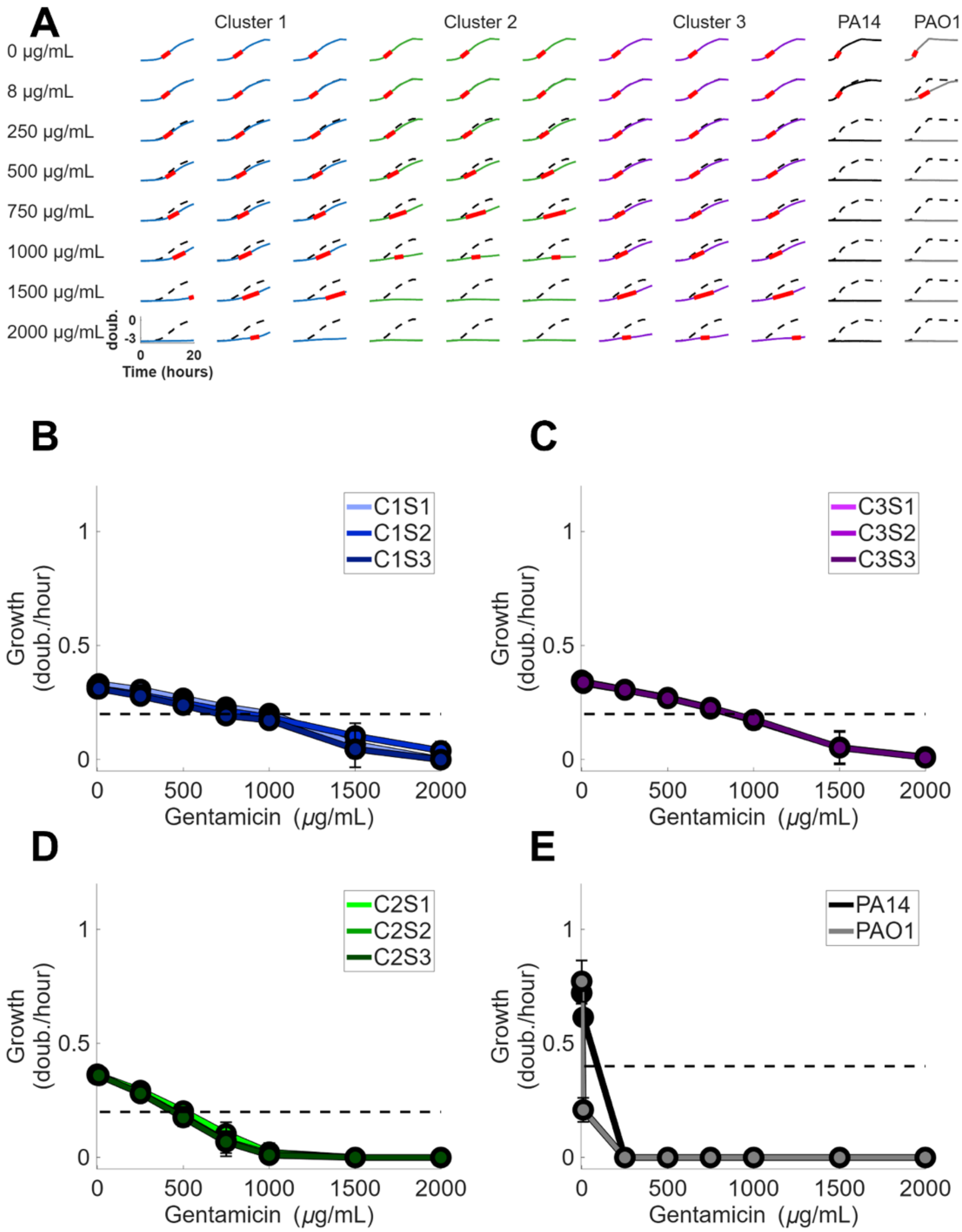

Figure S6: Calculation of gentamicin resistances from cell growth during a drug exposure. (A) Isolates from clusters 1-3, PA14, and PAO1 following exposure to a gradient of gentamicin, performed in three replicates. One representative plate is shown. Drug is added before the inoculation with cells at time = 0. The red line shows the steady-state growth following population recovery. The overlaid black dotted line represents growth in the absence of drug, which is the first row of each column. These growth curves were used to calculate the IC_{50} in **Fig. 4E** and MIC in **Fig S3C**. **(B-E)** Steady-state growth of each strain ("S") as a function of the gentamicin dose for **(D)** Cluster 1, **(E)** Cluster 2, **(F)** Cluster 3, and **(G)** lab strains. The horizontal line indicates half of the growth rate during exponential growth in the absence of drug. We calculate IC_{50} as the drug concentration that halves the maximum growth rate reached after exposure.

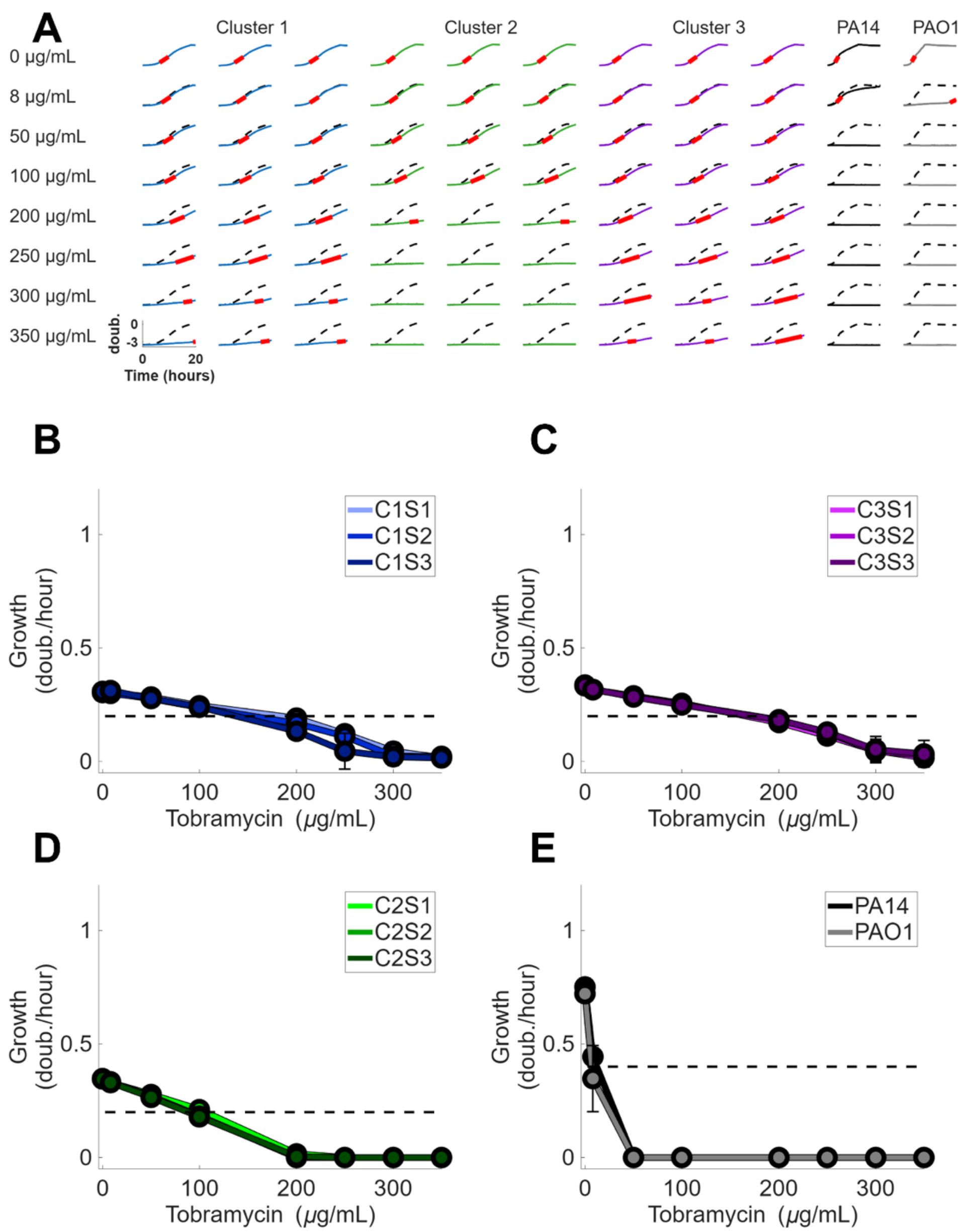

Figure S7: Calculation of tobramycin resistances from cell growth during a drug exposure. (A) Isolates from clusters 1-3, PA14, and PAO1 following exposure to a gradient of tobramycin, performed in three replicates. One representative plate is shown. Drug is added before the inoculation with cells at time = 0. The red line shows the steady-state growth following population recovery. The overlaid black dotted line represents growth in the absence of drug, which is the first row of each column. These growth curves were used to calculate the IC_{50} in **Fig. 4F** and MIC in **Fig S3D**. **(B-E)** Steady-state growth of each strain ("S") as a function of the tobramycin dose for **(D)** Cluster 1, **(E)** Cluster 2, **(F)** Cluster 3, and **(G)** lab strains. The horizontal line indicates half of the growth rate during exponential growth in the absence of drug. We calculate IC_{50} as the drug concentration that halves the maximum growth rate reached after exposure.

Table S1: Differentiation among populations by F_{ST}					
	Sample 1	Sample 2	F_{ST}*	Lower_CI	Upper_CI
Low	T1_Upper	T3_Upper	0.05756306	0.02821461	0.0879116
Differentiation	T2_Lower	T2_Upper	0.06930604	0.04509291	0.09359279
	T1_Middle	T3_Lower	0.18243142	0.11855742	0.24806492
	T3_Upper	T1_Middle	0.24706762	0.20207557	0.27897427
	T1_Middle	T2_Upper	0.29765606	0.20363133	0.38108922
	T1_Middle	T2_Middle	0.30246026	0.23163013	0.36313053
	T1_Middle	T2_Lower	0.30886207	0.26013064	0.35488344
	T3_Upper	T2_Upper	0.3444926	0.31021171	0.37612344
	T1_Middle	T1_Lower	0.34721102	0.30171511	0.38826122
	T1_Upper	T1_Middle	0.35896828	0.30817812	0.40070653
	T1_Upper	T1_Lower	0.373942	0.32859105	0.41161091
	T2_Lower	T3_Lower	0.37828169	0.30040402	0.43748612
	T1_Lower	T2_Upper	0.41288004	0.38007662	0.44909743
	T3_Upper	T1_Lower	0.42173126	0.36325002	0.47673472
	T2_Upper	T3_Lower	0.4274495	0.33070978	0.48652867
	T1_Upper	T2_Upper	0.45932355	0.40824949	0.50932731
	T1_Lower	T2_Lower	0.47194018	0.43345876	0.50558099
	T3_Upper	T3_Lower	0.47693078	0.43280834	0.51258619
	T3_Upper	T2_Lower	0.48875073	0.4196568	0.54703516
	T2_Middle	T3_Lower	0.53106071	0.37149024	0.64378159
	T1_Lower	T3_Lower	0.53391112	0.48682865	0.58438014
	T1_Upper	T3_Lower	0.55620329	0.49493579	0.60440812
	T1_Upper	T2_Lower	0.55719445	0.49992564	0.60587331
	T2_Lower	T2_Middle	0.60033561	0.50293784	0.6464499
	T1_Lower	T2_Middle	0.67878024	0.62660129	0.72359409
	T1_Upper	T2_Middle	0.69369026	0.62846685	0.73588708
	T2_Middle	T2_Upper	0.71333113	0.58153271	0.77909475
High	T3_Upper	T2_Middle	0.75936705	0.71671995	0.7964058

Table S1. Pairwise fixation index analysis (F_{ST}) and the lower and upper confidence interval (CI) are shown. Warmer colors indicate higher differentiation.

Gene	No. longitudinal studies	Presence in this study
algU	14	[green]
mucA	11	[green]
rpoN	11	[gray]
lasR	11	[green]
pagL	9	[gray]
mexA	9	[green]
mexZ	9	[gray]
mexB	8	[green]
migA	8	[green]
pvdS	7	[green]
ampD	6	[gray]
cbrB	6	[green]
exsA	6	[green]
rpoB	6	[gray]
gyrB	6	[gray]
pelA	5	[gray]
retS	5	[gray]
PA3728	5	[gray]
mexT	5	[gray]
spoT	5	[gray]
ampC	5	[gray]
algG	5	[green]
gyrA	5	[gray]
clpA	5	[gray]
pilJ	5	[gray]
mexY	5	[green]
nfxB	4	[gray]
oprD	4	[gray]
pmrB	4	[green]
fusA1	4	[green]
wbpM	4	[gray]
chpA	4	[green]
ftsI	4	[gray]
rpoC	4	[gray]
mexS	4	[green]

Table S2. This study has overlapping polymorphisms with prior CF *P. aeruginosa* longitudinal studies. The first column lists commonly mutated genes reported in 14 prior longitudinal studies of CF isolates, adapted from Fig. 1 of Camus *et al*. (1). The second column indicates whether a non-synonymous polymorphism in that gene was also observed in this study (green = detected; gray = not detected).

Supplement Reference

1. Camus L, Vandenesch F, Moreau K. 2021. From genotype to phenotype: adaptations of *Pseudomonas aeruginosa* to the cystic fibrosis environment. Microb Genom 7.
